# A selective defect in the glial wedge as part of the neuroepithelium disruption in hydrocephalus development in the mouse hyh model is associated with complete corpus callosum dysgenesis

**DOI:** 10.3389/fncel.2024.1330412

**Published:** 2024-02-21

**Authors:** Luis-Manuel Rodríguez-Pérez, Javier López-de-San-Sebastián, Isabel de Diego, Aníbal Smith, Ruth Roales-Buján, Antonio J. Jiménez, Patricia Paez-Gonzalez

**Affiliations:** ^1^Departamento de Fisiología Humana, Histología Humana, Anatomía Patológica y Educación Física y Deportiva, Universidad de Málaga, Malaga, Spain; ^2^Instituto de Investigación Biomédica de Málaga (IBIMA), Malaga, Spain; ^3^Departamento de Biología Celular, Genética y Fisiología, Universidad de Málaga, Malaga, Spain; ^4^Departamento de Anatomía y Medicina Legal e Historia de la Ciencia, Universidad de Málaga, Malaga, Spain

**Keywords:** dysgenesis of corpus callosum, agenesis of corpus callosum, hydrocephalus, glial wedge, indusium griseum glial cells, neuroepithelium, radial glial cells

## Abstract

**Introduction:**

Dysgenesis of the corpus callosum is present in neurodevelopmental disorders and coexists with hydrocephalus in several human congenital syndromes. The mechanisms that underlie the etiology of congenital hydrocephalus and agenesis of the corpus callosum when they coappear during neurodevelopment persist unclear. In this work, the mechanistic relationship between both disorders is investigated in the hyh mouse model for congenital hydrocephalus, which also develops agenesis of the corpus callosum. In this model, hydrocephalus is generated by a defective program in the development of neuroepithelium during its differentiation into radial glial cells.

**Methods:**

In this work, the populations implicated in the development of the corpus callosum (callosal neurons, pioneering axons, glial wedge cells, subcallosal sling and indusium griseum glial cells) were studied in wild-type and hyh mutant mice. Immunohistochemistry, mRNA in situ hybridization, axonal tracing experiments, and organotypic cultures from normal and hyh mouse embryos were used.

**Results:**

Our results show that the defective program in the neuroepithelium/radial glial cell development in the hyh mutant mouse selectively affects the glial wedge cells. The glial wedge cells are necessary to guide the pioneering axons as they approach the corticoseptal boundary. Our results show that the pioneering callosal axons arising from neurons in the cingulate cortex can extend projections to the interhemispheric midline in normal and hyh mice. However, pioneering axons in the hyh mutant mouse, when approaching the area corresponding to the damaged glial wedge cell population, turned toward the ipsilateral lateral ventricle. This defect occurred before the appearance of ventriculomegaly.

**Discussion:**

In conclusion, the abnormal development of the ventricular zone, which appears to be inherent to the etiology of several forms of congenital hydrocephalus, can explain, in some cases, the common association between hydrocephalus and corpus callosum dysgenesis. These results imply that further studies may be needed to understand the corpus callosum dysgenesis etiology when it concurs with hydrocephalus.

## 1 Introduction

The corpus callosum is the major interhemispheric bundle of commissural fibers in the brain, connecting the two brain hemispheres and permitting communication between the right and left sides of the brain. Alteration in the development of this structure can give rise to partial or complete agenesis, one of the most common malformations in the central nervous system (Diogo et al., 2021), with a prevalence of 1.8–3.3 per 10,000 livebirths (Morris et al., 2019; Pânzaru et al., 2022). Corpus callosum agenesis commonly coexists with other central nervous system pathologies, including interhemispheric cyst (Krupa and Bekiesinska-Figatowska, 2013). The common causes of corpus callosum agenesis are gene mutations related to axon guidance, ciliary development, cell adhesion, proliferation, and differentiation (Hofman et al., 2020). Corpus callosum agenesis can be present with arrested or progressive ventriculomegaly (Edwards et al., 2014; Masmejan et al., 2020; Hernandez et al., 2022; Pânzaru et al., 2022; Ahmed et al., 2023). However, cases of agenesis of the corpus callosum with isolated congenital hydrocephalus are rare (Paul et al., 2007; Verhagen et al., 2011; Hernandez et al., 2022). The explanation for the association of corpus callosum agenesis with hydrocephalus in some human syndromes is unknown. For instance, in syndromes related to defects in the L1 cell adhesion molecule, hydrocephalus is almost always present in the cases (Adle-Biassette et al., 2013). However, mice with knockout of the L1 do not develop hydrocephalus (Demyanenko et al., 1999).

The correct corpus callosum development needs the birth and specification of commissural neurons and proper axon guidance across the midline toward their final target in the contralateral hemisphere (Paul et al., 2007). In these events, sets of neurons and midline cell populations are needed: midline zipper glia, the subcallosal sling, the glial wedge, and the indusium griseum glia (Koester and O’Leary, 1994; Rash and Richards, 2001; Lindwall et al., 2007; Nishikimi et al., 2013; Suárez et al., 2014; Gobius et al., 2016). These populations have been described in humans (Lent et al., 2005; Ren et al., 2006; Jovanov-Milošević et al., 2009) and mouse embryos (Silver and Ogawa, 1983; Silver et al., 1993; Shu and Richards, 2001), and they are located rostrally to the lamina terminalis, in the midline, in continuity with the dorsal interhemispheric fissure (Silver et al., 1993). During the development, the deepening of the interhemispheric fissure results in the clefting of the dorsal lamina reuniens and the formation of the sulcus medianus telencephali medii. The two lips of this sulcus come in close approximation at the edge of the cortical plate in the corticoseptal boundary, and the fusion above the septal area in the midline forms the massa commissuralis (Jovanov-Milošević et al., 2009; Raybaud, 2010). The subcallosal sling and the midline zipper glia are the most likely equivalent of the massa commissuralis in human development at the tenth week of gestation (Rakic and Yakovlev, 1968; Barkovich and Norman, 1988). Primary cilia play a role in the morphogenesis of the telencephalon dorsoventral patterning and, therefore, the corticoseptal boundary and malposition of the guidepost cells. Thus, corpus callosum agenesis is common in ciliopathies (Laclef et al., 2015; Thomas et al., 2019).

Pioneering axons located in the cingulate cortex and projecting toward the contralateral cortex (Koester and O’Leary, 1994; Rash and Richards, 2001) delineate a pathway that is used by neocortical callosal axons to cross the telencephalic midline (Rash and Richards, 2001). The pioneering axons cross the midline and enter the contralateral hemisphere at E15.5 in mice (E, embryonic day), and from E16.5, they guide the subsequent callosal projecting axons. In human embryos, the pioneering axons cross at 12–13th gestational week (GW) (Rakic and Yakovlev, 1968), and the corpus callosum is formed between GW 14–21st (Malinger and Zakut, 1993; Kier and Truwit, 1997; Achiron and Achiron, 2001; Ren et al., 2006).

Subcallosal sling was initially identified as a glial cell population that migrated from the ventricular zone, which would be implicated in developing the midline structures, including the corpus callosum (Silver and Ogawa, 1983; Silver et al., 1993). Later results proved that subcallosal sling is a migratory population of developing neurons (Shu et al., 2003a). The role of the subcallosal sling in axonal guidance and its role in dysgenesis of the corpus callosum has been demonstrated and described in mice and humans (Silver et al., 1982; Silver and Ogawa, 1983; Shu et al., 2003a; Ren et al., 2006). Neuron sling participates in the callosal axon guidance through the midline but does not participate in the pioneering axon guidance (Shu et al., 2003a).

The glial wedge and the indusium griseum glial cells are ventral and dorsal to the corpus callosum, respectively (Shu and Richards, 2001). The glial wedge cell bodies are located in the ventricular zone of the lateral ventricles, at the corticoseptal boundary, and send long radial-glial-like processes toward the brain midline. The glial wedge and indusium griseum glial cells are guidepost cells that provide guidance cues that prevent axons from leaving the tract and entering adjacent structures (Richards et al., 2004; Lindwall et al., 2007). The glial wedge and the indusium griseum glial cells produce molecules such as the chemorepellents Slit2 (Silver and Ogawa, 1983; Booth et al., 2000; Hidalgo and Booth, 2000; Shu and Richards, 2001; Shu et al., 2003c; Lent et al., 2005; Ren et al., 2006; Lindwall et al., 2007; Unni et al., 2012), Wnt5a (Keeble et al., 2006), and Draxin (Islam et al., 2009), or chemoattrant Netrin1 (Fothergill et al., 2014; Ahmed and Shinmyo, 2021). Thus, these cells generate a path that is used by pioneering and callosal axons to cross the midline during corpus callosum development (Silver and Ogawa, 1983; Booth et al., 2000; Hidalgo and Booth, 2000; Shu et al., 2003c; Barresi et al., 2005; Lent et al., 2005; Ren et al., 2006). The corpus callosum is not fully developed when the glial wedge cells are experimentally excised (Shu and Richards, 2001). In the mouse, the glial wedge cells begin to be formed at E13 (Shu et al., 2003b), and it is fully developed at E15 (Shu and Richards, 2001), just before the projection of the pioneering and callosal axons (Shu et al., 2003c).

In conclusion, the ultimate cause of partial or complete corpus callosum dysgenesis is the alteration of the necessary cell populations derived from the neuroepithelium/radial glia (Gobius et al., 2016). However, the reason why these populations are defective may vary depending on the neurodevelopmental disorder. Fetal cerebral ventriculomegaly linked to neuroepithelium/radial glia disruption appears to be a relevant feature in the origin of some forms of congenital hydrocephalus (Jiménez et al., 2001; Domínguez-Pinos et al., 2005; Ferland et al., 2009; Sival et al., 2011; Rodríguez et al., 2012; Guerra et al., 2015; Duy et al., 2023). This study aims to investigate the defect that underlies the corpus callosum dysgenesis in a model of congenital hydrocephalus to discern if ventricular dilatation was the linking nexus between both pathologies. For this purpose, we have used the hyh mutant mouse (*hy*drocephalus with *h*op gait) (Bronson and Lane, 1990). In the hyh mouse, the *Napa* gene that encodes the soluble N-ethylmaleimide-Sensitive factor (NSF) Attachment Protein, alpha-SNAP, is mutated (Chae et al., 2004; Hong et al., 2004). Hyh mice carry a hypomorphic missense mutation in the *Napa* gen that encodes for protein αSNAP, which is involved in SNAP receptor (SNARE)-mediated vesicle fusion in many cellular contexts. The hypomorphic mutation of the *Napa* gene provokes an unstable mRNA and therefore low levels of protein αSNAP; however, it does not disrupt the protein *per se* (Chae et al., 2004). The residual protein in hyh mutants is partially functional, as a targeted null mutation of *Napa* is embryonically lethal (Chae et al., 2004). αSNAP regulates protein trafficking (Clary et al., 1990; Püschel et al., 1994; Yoon and Munson, 2018). It has been described that partial loss of αSNAP disrupts its ability to bind to its target, but does not disrupt its cellular functions, what suggests a dose-related role in apical trafficking (Chae et al., 2004). The exact molecular pathways by which this mutation operates in the defective neuroepithelium are not known.

However, the consequence of this mutation is well described: an alteration in neural cell fate (Chae et al., 2004) and an alteration of the neuroepithelium/ventricular epithelium following a well-defined program (Jiménez et al., 2001). After neuroepithelial cells transition to radial glial cells, as cells start to differentiate and mature, the denudation of the ventricular epithelium starts to be detected along the ventricular walls (Jiménez et al., 2001; Chae et al., 2004). The defective program of cell differentiation existing in the neuroepithelium/radial glial cells of the hyh mutant mouse has been profusely detailed (Jiménez et al., 2001; Wagner et al., 2003; Bátiz et al., 2006; Paez et al., 2007). This defective program of cell differentiation affecting ventricular epithelium follows a temporo-spatial pattern that progresses accordingly to the developmental patterning of the anterolateral neural plate (telencephalon) during prosencephalic regionalization: caudorostral linear axis, mediolateral linear axis and pallial-subpallial limit radial axis (Rubenstein et al., 1998; Puelles et al., 2000; Jiménez et al., 2001; Taverna et al., 2014; Puelles, 2017; Garcia-Calero and Puelles, 2020).

In the hyh mouse, hydrocephalus is mild during the development and postnatally becomes severe after obstruction of the cerebral aqueduct (Pérez-Fígares et al., 1998; Wagner et al., 2003; Bátiz et al., 2006; Paez et al., 2007). The hyh mouse exhibits dysgenesis of the corpus callosum (Bátiz et al., 2006; Paez et al., 2007) and develops hydrocephalus with an interhemispheric cyst (Bronson and Lane, 1990; Pérez-Fígares et al., 1998) which models human congenital hydrocephalus (Barkovich et al., 2001; Bátiz et al., 2006; Paez et al., 2007).

In the present work, we have found that the pioneering axons arose from cingulate neurons in the hyh mouse but failed to cross the midline. Instead of turning toward the contralateral hemisphere, the pioneering axons turned toward the ipsilateral ventricle. We have also found that glial wedge cells in the hyh mouse were selectively affected by the defective developmental program of the ventricular zone at the moment of the projection of the pioneering axons. We have finally found that these defects in the ventricular zone development occur before any ventricular dilatation. In this way, we have concluded that the corpus callosum dysgenesis in a model of congenital hydrocephalus is caused by a radial glial cell defect that induces a problem in axonal guidance during development. The radial glial cell defect also causes the hydrocephalus in this model of congenital hydrocephalus. Therefore, the defect in the radial glial cell population is the mechanism underlying these two key pathologies. Defects in the normal development of the neuroepithelium and radial glial cells integrating the ventricular zone are found in different cases of human congenital hydrocephalus with dysgenesis of the corpus callosum (Jin et al., 2020). The results presented in this work demonstrate the need to review the origin of the dysgenesis of the corpus callosum in cases of human congenital hydrocephalus with defects in the neuroepithelium/radial glial cells to obtain the correct prognosis and to determine appropriate therapy for the patient.

## 2 Materials and methods

### 2.1 Animals

Hyh mutant mice (B6C3Fe-a/a-hyh/J) were obtained from The Jackson Laboratory (Bar Harbor, ME) and bred into a colony at the University of Malaga, Spain. Housing, handling, care and processing of animals were carried out according to European and Spanish laws (DC 86/609/CEE and RD 1201/2005). According to current legislation, experimental procedures (protocol 28-08-15-302) were approved by the Institutional Animal Care and Use Committee of the University of Malaga (CEUMA, Spain 65-2019-A) and the Regional Government Council (Junta de Andalucía, Spain). Gestational day 0.5 (E0.5) was designated when a vaginal plug was evident in a pregnant female. Pregnant dams at gestational days between E15.5 and E18.5 were sacrificed, and the embryos were removed by laparotomy. All animals used in this work were genotyped as described by Bátiz et al. (2009). The double homozygous mice for the mutated *Napa* gene that codifies α-SNAP protein [*Napa*(-/-)] were assigned as hyh mice, and normal mice [*Napa*(+/+)] were set as wild-type (wt). Heterozygous mice for α -SNAP were used only to obtain homozygous hyh embryos.

### 2.2 Hematoxylin-eosin staining, immunocytochemistry, and immunofluorescence

Brains of hyh and wt fetuses (E15.5 to E18.5) and mice at 1 to 4 postnatal days (P1 to P4) were processed for hematoxylin-eosin staining, immunocytochemistry, and immunofluorescence, using frontal and sagittal sections. Sampling was as follows: E14.5, 10 hyh and 13 wt; E15.5, 21 hyh and 21 wt; E16.5, 10 hyh and 10 wt; E17.5, 12 hyh and 12 wt; E18.5, 8 hyh and 8 wt; P1, 8 hyh and 8 wt; P2, 8 hyh and 8 wt; P3, 8 hyh and 8 wt; and P4, 19 hyh and 14 wt. Whenever possible, mice belonging to the same litter were processed simultaneously to compare hyh and wt mice at the same age. Embryos and early postnatal mice were euthanized by decapitation. Then, the brains were dissected and fixed for 2–4 days in Bouin fixative solution (room temperature) or 4% paraformaldehyde in 0.1 M phosphate buffer, pH 7.4 (4°C). Bouin-fixed brains were embedded in paraffin, and sections (10 μm thick) were obtained. Paraformaldehyde-fixed brains were sliced with a vibratome (50 μm thick sections).

Paraffin and vibratome sections were processed for immunocytochemistry/immunofluorescence using the following primary antibodies: rabbit polyclonal anti-Calretinin (1:3000 dilution, 7699/4, Swant Antibodies, Bellinzona, Switzerland); mouse monoclonal anti-βIII Tubulin (1:500 dilution, G712A, PROMEGA. WI, USA); mouse monoclonal anti-GAP-43 (1:2000 dilution, G9264, Clone GAP-7B10, Sigma-Aldrich, St. Louis, MO, USA); rabbit polyclonal anti-GFAP (1:1000 dilution, Z0334, Agilent Dako, Santa Clara, CA, USA); mouse monoclonal anti-GFAP (1:1000 dilution, G-A-5, Sigma-Aldrich, St. Louis, MO, USA); mouse monoclonal anti-NCAM (undiluted, 5B8 clone, Developmental Studies Hybridoma Bank, DSHB, Iowa City, IA); rabbit monoclonal anti-NeuN (1:500 dilution, ab177487, Abcam, Cambridge, UK); mouse monoclonal anti-Nestin (1:200 dilution, clone Rat-401, DSHB); mouse monoclonal anti-PCNA (1:200 dilution, MAB424, Clone PC10, Sigma-Aldrich, St. Louis, MO, USA); and rabbit monoclonal anti-Slit2 (1:500 dilution, ab134166 Abcam, Cambridge, UK). Appropriate AlexaFluor (Molecular Probes, Carlsbad, CA) or biotin-labeled secondary antibodies (Dako, Glostrup, Denmark) were used for immunofluorescence/immunocytochemistry. Biotin-labeled secondary antibodies were detected using ExtrAvidin-peroxidase (Sigma-Aldrich). Histochemistry for peroxidase was developed using 3,3′-diaminobenzidine tetrahydrochloride (DAB, Sigma-Aldrich) as the electron donor. Ammonium nickel sulfate was added to intensify the DAB reaction in some sections. The antibodies against Slit2 were used only in vibratome-sliced sections. In the paraffin sections, heat-induced antigen retrieval was performed in 50 mM citrate buffer at pH 6.0 before immunolabeling. The omission of the incubation in the primary antibody was used as a control of the immunoreaction.

### 2.3 *In situ* hybridization

Embryos at E14.5 to E18.5 and newborns at 1 to 7 days postnatal age (P1 to P7) were processed for non-radioactive *in situ* hybridization using digoxigenin-labeled riboprobes. Sampling was as follows: E14.5, 19 hyh and 20 wt; E15.5, 20 hyh and 20 wt; E16.5, 15 hyh and 15 wt; E17.5, 15 hyh and 15 wt; E18.5, 15 hyh and 15 wt; P1, 15 hyh and 15 wt; P4, 12 hyh and 12 wt; P7, 12 hyh and 12 wt. Mouse embryos were fixed by immersion or vascular perfusion with 4% buffered paraformaldehyde. Then, they were kept at 4°C until dehydration in methanol/PBT (PBS, 0.1% Tween-20). After rehydration, the telencephalon was dissected and immersed into a gelatin/albumin solution (30% albumin and 0.5% gelatin in 0.1 M phosphate buffer, pH 7.3) containing 1.25% glutaraldehyde. Vibratome sections (200 μm thickness) were obtained, dehydrated in methanol/PBT, and kept at −20°C overnight. Single *in situ* hybridization was performed for whole-mount preparations (Wilkinson, 1992). Probes were labeled with digoxigenin-UTP (Boehringer, Ingelheim, Germany) using the Riboprobe Gemini System II Kit (Promega, Madison, WI). Labeled probes were added to the hybridization buffer at 1–2 mg/ml concentration. Sections were hybridized at 70°C overnight. An anti-digoxigenin-alkaline phosphatase-coupled antibody was used to detect the probes (1:2,000; 11-093274916, Boehringer). Alkaline phosphatase activity was revealed using NBT/BCIP as a substratum. In all cases, the sense probes did not show an unspecific signal. Stained sections were flat-mounted on poly-L-Lysine-treated slides with 80% glycerol in PBT. Riboprobes for Netrin1 (de Diego et al., 2002), Tag1 (transient axonal glycoprotein) and Tbr1 (T-box, brain, 1) were produced as described by Bulfone et al. (1995) and Denaxa et al. (2001).

### 2.4 DiI-tracing of callosal axons in fixed brains

Brains of mice at P1 (3 wt and 3 hyh) were dissected out and fixed in 4% paraformaldehyde in 0.1 M phosphate buffer, pH 7.4 at 4°C for 4 days. After fixation, brains were washed out in PBS 0,1 M pH 7,4. A small crystal of DiI (1,1-dioctadecyl-3,3,3′,3′-tetramethylindocarbocyanine perchlorate; D-3911, Molecular probes) was placed in the frontal cortex of both hemispheres of whole dissected brains, which were then put in an oven at 37°C for 3 weeks. After incubation, 300 μm thick slices with a vibratome and photographed under a fluorescence microscope.

### 2.5 DiI-tracing of the pioneering axons in organotypic culture of brain slices

Brains of embryos at E14.5 (9 wt and 9 hyh) and E15.5 (9 wt and 12 hyh) were dissected out under immersion into ice-cold L15/F12 medium, pH 7.4 (50% Leibovitz’s L15 medium, Life Technologies, Carlsbad, CA; 50% F12 nutrient mixture, Life Technologies). Then, they were immersed into 3% low-melting agarose (Gellyphor, Euroclone, Siziano, Italy) to obtain frontal 300 μm thick slices with a vibratome. Sections were collected in ice-cold L15/F12 medium and transferred onto filter inserts (31.5 μm diameter, 0.4 μm pore size; Merck Millipore, Billerica, MA) in dishes containing 1 ml of sterile-filtered medium with serum (DMEM containing 8% F12 nutrient, 1% N-2 supplement, 15% glucose, 1% penicillin-streptomycin P0781 Sigma-Aldrich, 0.5% L-glutamine, and 5% heat-inactivated fetal bovine serum (F7524, Sigma-Aldrich). After 1 h in a sterile incubator (37°C, 5% CO_2_), the medium was substituted for neurobasal medium, pH 7.4 (21103049, Gibco), containing 2% B27 supplement (17504044, Gibco), 15% glucose, 1% penicillin-streptomycin (P0781, Sigma-Aldrich), 0.5% L-glutamine (Sigma-Aldrich), and 2.5% heat-inactivated fetal bovine serum (F7524, Sigma-Aldrich). Immediately after this step, a tiny crystal of DiI was placed into the cingulate cortex of the brain slices, and they were incubated in sterile conditions (37°C, 5% CO2) for 48 h. Finally, slices were fixed in 4% buffered paraformaldehyde at 4°C for 24 h. Selected fixed slices were then processed for immunofluorescence using the anti-GFAP antibody.

## 3 Results

### 3.1 The development of the corpus callosum is defective and precedes ventricular dilation

**FIGURE 1:**
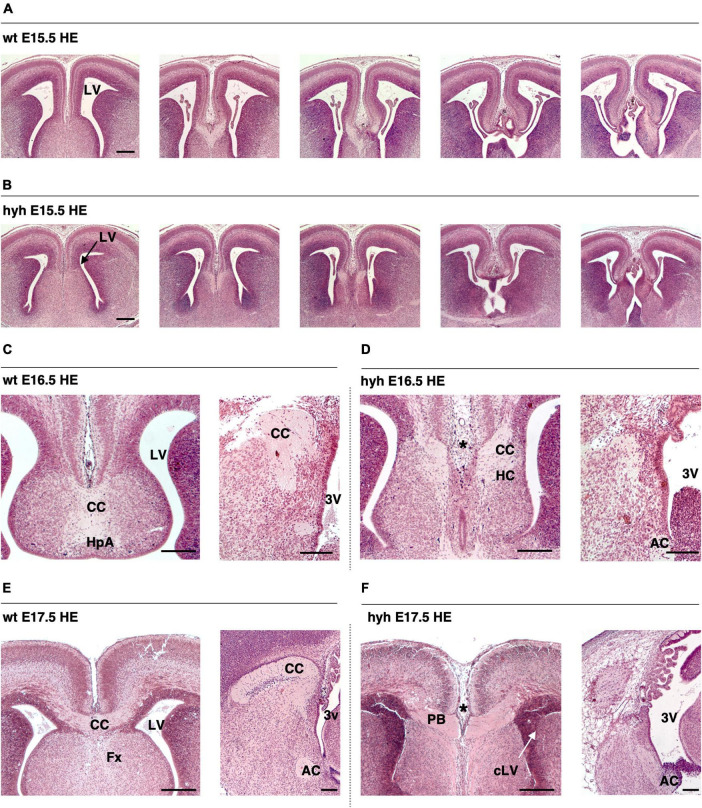
The corpus callosum development is affected in the hyh mouse. Frontal sections of the telencephalic lobes of the brain of wild-type (wt) **(A,C,E)** and hyh mutant (hyh) mice **(B,D,F)** at 15.5 **(A,B)**, E16.5 **(C,D)**, and E17.5 **(E,F)**. **(C–F)** Midline sagittal sections are shown on the right. Hematoxylin-Eosin staining (HE). At E17,5, callosal projections can be observed in the normal mouse crossing the midline to form the corpus callosum **(E)**. The callosal projections fail to cross the midline at E16.5 and E17.5 in the hyh mouse [asterisks in panels **(D,F)**]. In the hyh mutant mouse, from E15.5 to E17.5, lateral ventricles are not enlarged **(B,D,F)**; in contrast, they appear collapsed by E17.5 **(F)**. AC, Anterior Commissure; CC, Corpus Callosum, Fx, Fornix; HC, Hippocampal Commissure; LV, Lateral Ventricle; cLV, collapsed Lateral Ventricle; PB, Probst Bundles; 3V, Third Ventricle. Scale Bars: 200 μm.

The corpus callosum development and the ventricular dilatation evolution from E14.5 to P2 were studied by Hematoxylin-Eosin staining (Figures 1A–F). In the wt mouse, the corpus callosum was detectable at E16.5 (Figure 1C) and appeared strongly developed at E17.5 (Figure 1E). The anterior commissure was present in the hyh mouse at these stages in 100% of the studied mice, but the corpus callosum fibers were not crossing the midline (Figures 1D, F).

In the hyh mouse, hydrocephalus was still mild between E14.5 and E16.5, when the corpus callosum should have been developed (Richards et al., 2004). Remarkably, the lateral ventricles were not enlarged, or even they collapsed during this period, and no cyst of the third ventricle between the telencephalic hemispheres was perceptible (Figures 1A–F). The most of the hyh mutant animals present hippocampal commissure defects, the fornix is formed but does not seem to cross (Figures 1A–F).

Analyzing the ventricular dilatation at the age at which development of the corpus callosum occurs, we could observe that brain ventricle dilatation could not be the reason for the lack of axonal projections of the corpus callosum in the midline (Figures 1A–F). The neurons involved in the growth and guidance of the callosal axons and the glial cell populations involved in the development of the corpus callosum were analyzed to understand what could be causing the dysgenesis of the corpus callosum in the hyh mouse.

### 3.2 The cortical regions implied in the corpus callosum development are developed in the hyh mutant mouse

First, we studied if the cortical regions where the callosal and pioneering neurons reside showed alterations that could explain the corpus callosum dysgenesis detected in the hyh mutant animals. The soma of the neurons that generate the pioneering axons are in the ipsilateral presumptive cingulate cortex at E14 (Koester and O’Leary, 1994; Rash and Richards, 2001). The soma of the neurons that project the callosal axons are located in the ipsilateral frontal cortex at E14 (Koester and O’Leary, 1994). We studied these cortical regions at E14.5 and E15.5.

**FIGURE 2:**
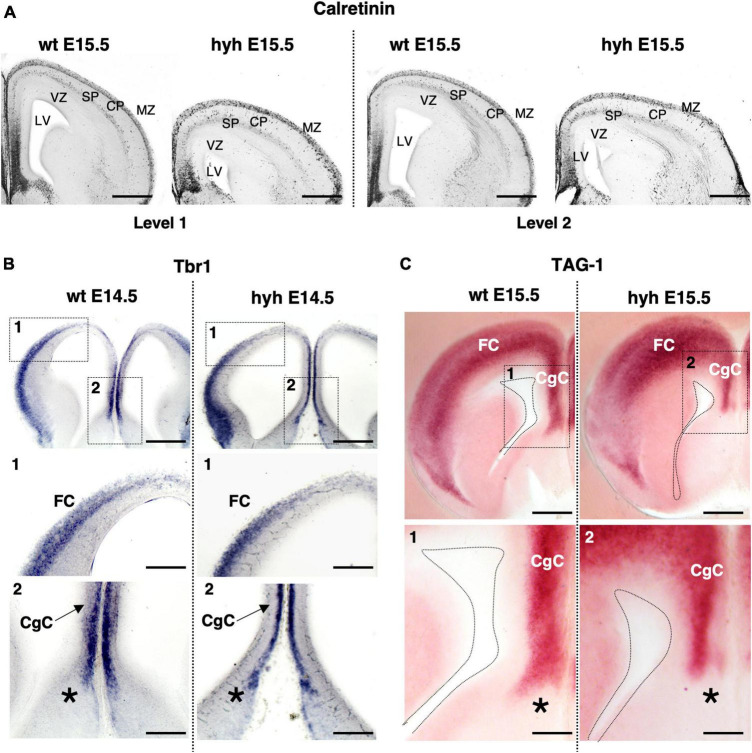
The cortical regions of the corpus callosum development. **(A)** Immunostaining against Calretinin protein in frontal sections of the brain from wild-type (wt) (left) and hyh mutant (right) embryos at E15.5, at two different rostrocaudal levels (Level 1 and Level 2). *In situ* hybridization for Tbr1 **(B)** and Tag1 mRNA **(C)** in frontal sections of the telencephalic lobes from the brain of wild-type (wt) (left) and hyh mutant (right) mice at E14.5 and E15.5. Frames 1 and 2 and details from wt and hyh mice in panels **(B,C)** are shown at the bottom. CgC, Cingulate Cortex; CP, Cortical Plate; FC, Frontal Cortex; LV, Lateral Ventricle; MZ, Marginal Zone; SP, Subplate; VZ, Ventricular Zone. The asterisk shows the limit between septal pallial and subpallial areas. Scale Bars: **(A)**, 200 μm; **(B)**, 200 μm; [Frames 1 and 2 in panel **(B)**], 100 μm; **(C)**, 200 μm; [Frames 1 and 2 in panel **(C)**], 100 μm.

Hematoxylin-Eosin staining at E15.5 shows a general size reduction in the mutant animal concerning the control but does not show any evident cortical alteration (Figure 1). Cortical structure was also studied with antibodies against Calretinin, a multifunctional protein expressed in the developing and adult cortex (González-Gómez and Meyer, 2014; Laclef and Métin, 2018; Ahmed and Shinmyo, 2021). Staining for Calretinin expression shows maintenance of the main cortical structure in the mutant animals at E15.5 (Figure 2A).

Additionally, the cingulate and frontal cortices were studied by *in situ* hybridization for Tbr1 mRNA and Tag1 mRNA. Correct expression in cortical layers of Tbr1 is necessary for proper corpus callosum development (Bulfone et al., 1995; Crespo et al., 2022). Tag1 protein is an axonal adhesion molecule expressed in cortical neurons projecting efferent axons, both callosal and pioneering axons (Kastriti et al., 2019). Neurons located in the cingulate cortex and frontal cortex at E14.5 showed positive signals for Tbr1 mRNA probes in both wt and mutant animals (Figure 2B). Probes for Tag1 mRNA hybridized in both wt and hyh mice at E15.5 with the same pattern (Figure 2C).

### 3.3 The ability of the neurons to produce the callosal axonal projections is not lost in the mutant hydrocephalic animals

**FIGURE 3:**
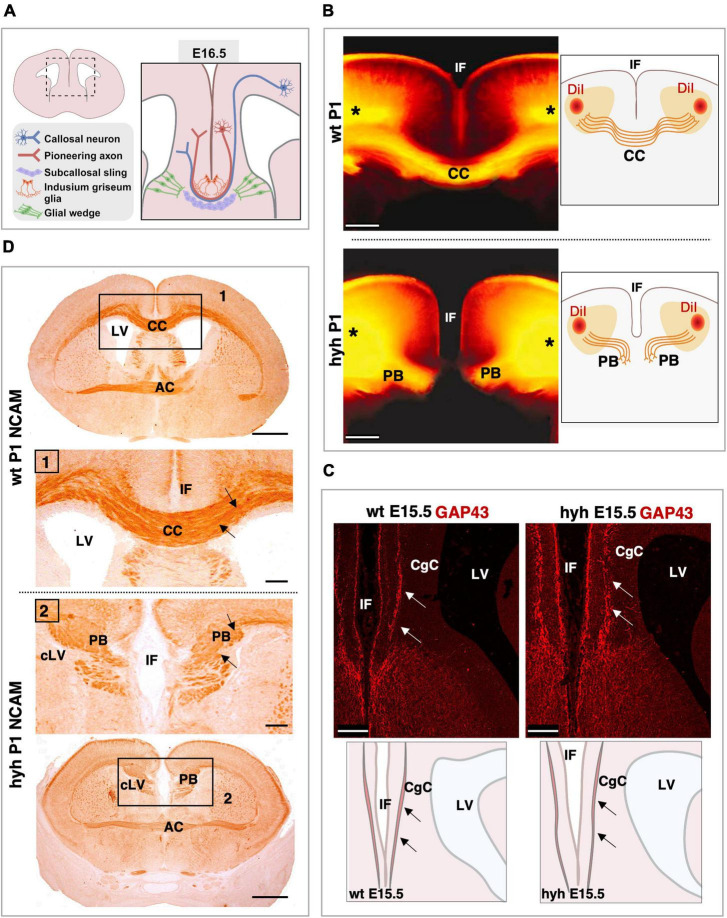
Callosal axonal projections. **(A)** Scheme of a frontal section of the brain from a normal mouse embryo at E16.5 representing the populations implicated in the correct corpus callosum development. **(B)** DiI-tracing experiment to label the callosal fibers in fixed mouse brains at P1. The positions where the DiI crystals were placed are shown (asterisks). The callosal fibers can be detected crossing the midline in the wild-type (wt) mouse (Top). However, in the hyh mutant mouse (Bottom), callosal axons do not cross the midline and form Probst Bundles (PB). Schemes on the right depict where DiI crystals were placed and the path that callosal axons follow. **(C)** Frontal sections of wt (Left) and hyh (Right) mutant mice brain at E15.5 showing GAP-43 immunostaining. The main anatomical structures detected in the pictures are depicted in schemes on the bottom. In wt and hyh mutant mice, pioneering axons are GAP43-positive (arrows) and do not show any anatomical alteration in the hyh mouse. **(D)** Frontal sections of mice brains at P1 showing NCAM immunolabeling. In wt (Top) and hyh mutant (Bottom) mice, callosal axons are NCAM positive (arrows). Frames 1 and 2 are shown in detail. AC, Anterior Commissure; CC, Corpus Callosum; CgC, Cingulate Cortex; IF, Interhemispheric Fissure; LV, Lateral Ventricle; cLV, collapsed Lateral Ventricle. Scale Bars: **(B)**, 200 μm; **(C)**, 100 μm; **(D)**, Panoramic, 100 μm; Frame 1 and Frame 2, 50 μm.

Knowing that cortical areas involved in corpus callosum development were not disrupted in the hyh mutant animals, the neurons involved in the generation and guidance of the callosal axons, as well as the glial cell populations necessary for the correct development of the corpus callosum, were analyzed: callosal neurons, pioneering axons, subcallosal sling, indusium griseum glial cells, and glial wedge cells (Figure 3A). The elongation the callosal and pioneering axons were first studied (Figures 3B–D).

The tissue was analyzed with Hematoxylin-Eosin staining from 16.5 to P1. The axonal projections incapable of crossing the midline were detected from 16.5. These axonal projections were accumulated in the midline area, generating the Probst bundles (Figures 1E, F).

To verify that the Probst bundles were generated by callosal axons from the frontal cortex in the mutant animals and not from hippocampal cortical neurons, we used the DiI-axonal trace labeling technique at P1. In both wt and mutant hyh mice, DiI crystals were placed in the frontal cortex of fixed brains, in the cortical region where the callosal neurons are located (Figure 3B). DiI was allowed to diffuse by the axons for 2 weeks, and the axonal projections were studied. Results showed that the Probst bundles existing at P1 were generated by the axonal projections emerging from the callosal neurons from the frontal cortex (Figure 3B).

Then, we analyzed if the pioneering axons from the cingulate cortex could elongate projections to help guide the callosal axons through the midline during the interhemispheric cross. For this purpose, we studied the midline area with GAP-43 (growth-associated protein), a marker for growing axons (Oestreicher et al., 1997). At E15.5 and in this specific area of the cortex, this marker should only label pioneering axons as the callosal axons have not yet arrived at the cingulate cortex (Rash and Richards, 2001; Suárez et al., 2014). Results showed that pioneering axons are present in both wt and hyh mutant mice in the cingulate cortex at 15.5, and no differences were detected between mutant and normal mice (Figure 3C).

Finally, NCAM expression was studied. NCAM is one of the main proteins involved in the ability of the neurons to generate cell-to-cell interactions and cell-matrix interactions that allow axons and neurons to properly elongate or migrate (Seki and Arai, 1993). NCAM expression from 16.5 to P1 did not show differences between normal and mutant animals (Figure 3D). Therefore, even if the axons were not able to cross the midline, the ability of these axons to establish cell-to-cell interactions as well as cell-matrix interactions appeared not to be defective in the hyh mutant mouse.

### 3.4 The subcallosal sling neurons are present in the mutant animals

Subcallosal sling cells are neurons involved in correct corpus callosum development (Shu et al., 2003a). No specific marker is available for this population (Shu et al., 2003a); thus, its presence was studied with NeuN, a specific marker for neurons.

**FIGURE 4:**
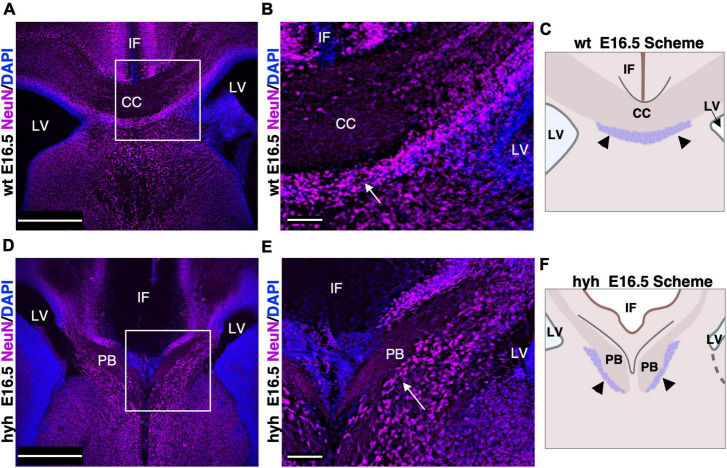
Subcallosal sling. **(A)** Frontal section of the wild-type mouse (wt) brain at E16.5 with a frame detailed in panel **(B)**. **(B)** The subcallosal sling cells positive to NeuN (arrows) are located ventrally to the callosal axons crossing the midline. **(C)** Scheme depicting the position of the callosal axons and subcallosal sling (arrowheads) in the wt mouse at E16.5. **(D)** Frontal section of the hyh mutant mouse brain at E16.5 with a frame detailed in panel **(E)**. **(E)** A group of NeuN positive cells (arrows) ventral to the Probst Bundles (PB) can correspond to the subcallosal sling in the hyh mouse. **(F)** Scheme depicting the displaced position with respect to the control of the callosal axons forming the PB and subcallosal sling (arrowheads) in the hyh mutant mouse. CC. Corpus Callosum, IF, Interhemispheric Fissure; LV, Lateral Ventricle. Scale Bars: **(A,C)**, 350 μm; **(B,D)**, 90 μm.

In the wt mouse at E16.5, the neurons of the subcallosal sling were detected with NeuN immunolabeling (Figures 4A–C). In the hyh mouse, these NeuN positive cells were also present and located correctly under the callosal fibers, which in this case are generating the uncrossing Probst bundles (Figures 4D–F). However, the subcallosal sling is not occupying the midline as the corpus callosum never cross the midline bundles (Figures 4D–F).

### 3.5 The glial wedge cells are not developed in the hyh mutant mouse

**FIGURE 5:**
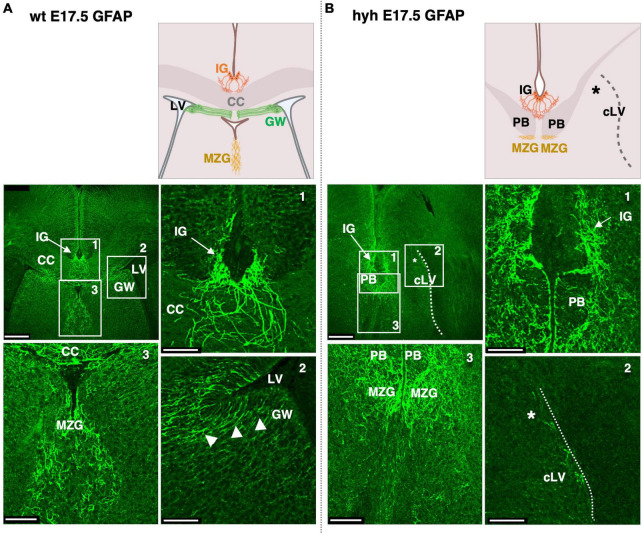
Indusium griseum glial and glial wedge cells. Frontal sections of wild-type (wt) **(A)** and hyh **(B)** mice brain at E17.5 with GFAP immunolabeling. On the top are panoramic views and schemes depicting the position of relevant anatomical structures. On the bottom the framed areas on the top are shown in detail. The Glial Wedge cells (GW), including their basal projections, can be detected as GFAP-positive in the wt mouse (arrowheads) but absent (asterisk) in the wall of the collapsed lateral ventricle (cLV, dashed line) of the hyh mouse. Midline zipper glia (MZG) is detected under the Probst Bundles (PB). CC, Corpus Callosum; IG, Indusium Griseum Glial Cells; LV, Lateral Ventricle. Scale Bars: **(A,B)**, 250 μm; (Frames 1, 2, 3), 65 μm.

We next analyzed the glial populations responsible for the guidance of callosal axons in their midline cross, the indusium griseum glial cells and glial wedge cells. These cells were studied at E17.5 by GFAP immunostaining. The indusium griseum glial cells were present in wt and hyh mice (Figures 5A, B). In contrast, glial wedge cells were not detected in the hyh mice (Figures 5A, B).

### 3.6 There is a disruption of the ventricular zone development that affects the glial wedge cells in the hyh mutant mouse

**FIGURE 6:**
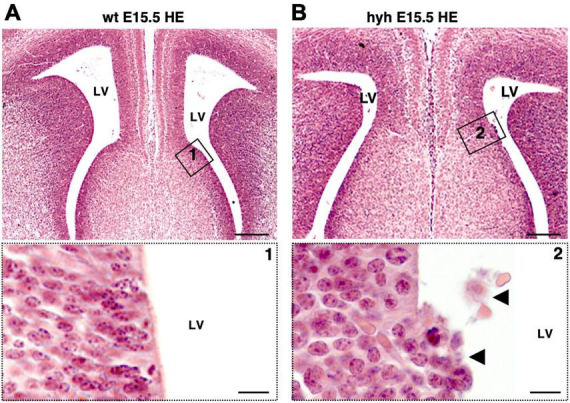
Disruption on the glial wedge cells area in the hyh mouse. Frontal sections of wild-type (wt) **(A)** and hyh mutant **(B)** mice brain at E15.5 stained with Hematoxylin-Eosin (HE). Details of framed areas 1 and 2 are shown at the bottom. In the hyh mutant mouse, detaching cells can be observed in the area of the glial wedge cells (arrowheads). LV, Lateral Ventricle. Scale Bars: **(A,B)**, 200 μm; (Frames 1, 2), 10 μm.

The absence of glial wedge cells in the hyh mutant mouse made it necessary to investigate the development of the ventricular zone region corresponding to glial wedge cells. The ventricular zone area containing the glial wedge cells was examined from E14.5 to E18.5 with Hematoxylin-Eosin staining. Results showed that, at E15.5, the ventricular zone region corresponding to glial wedge cells was disrupted in the hyh mutant mouse (Figures 6A, B).

**FIGURE 7:**
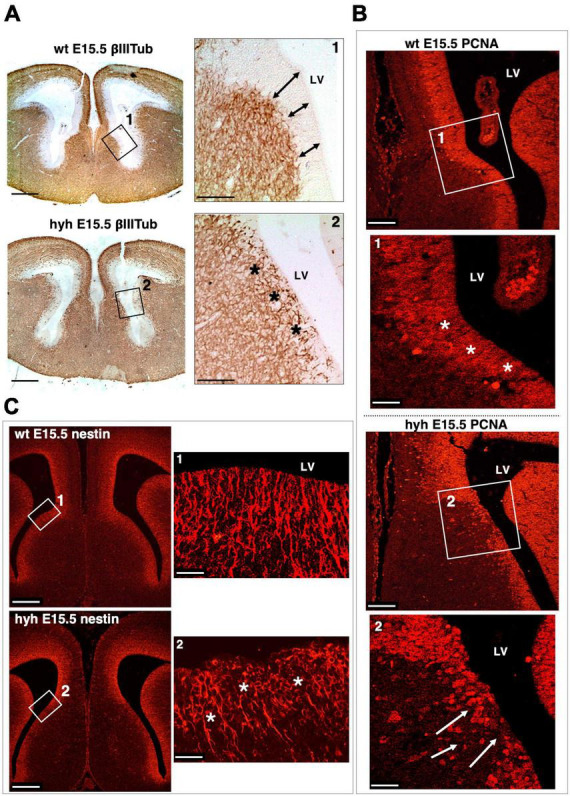
Ventricular zone alteration of the glial wedge area in the hyh mouse. **(A)** Frontal sections of wild-type (wt) (top) and hyh (bottom) mice brain at E15.5 immunostained with anti-βIII-Tubulin. Framed areas 1 and 2 are shown in detail on the right. This area corresponds to the region where the glial wedge cells will appear. In the wt mouse, the βIII-Tubulin-positive reaction appears under the ventricular zone (Frame 1). The ventricular zone is negative to βIII-Tubulin and presents well-defined borders (arrows). In the hyh mutant mouse (Frame 2), the βIII-Tubulin-positive reaction appears to reach the ventricular surface, invading the ventricular zone (asterisks). **(B)** Frontal sections of the wt and hyh mutant (hyh) mice brain at E15.5 stained with anti-PCNA. Proliferation in the ventricular zone area (PCNA-positive cells) is observed. Frame 1 shows a detail corresponding to the region where the glial wedge cells will appear. The ventricular zone in this area of the wt mouse is highly positive for PCNA antigen (asterisks). In frame 2, the ventricular zone is negative for PCNA in the hyh mouse (arrows). **(C)** Frontal sections of the wt and hyh mutant mice brain at E15.5 stained with anti-Nestin to label the radial glial cells of the ventricular zone in the region where the glial wedge cells will appear. Frame 1 shows the ventricular zone radial glial cells and their basal projections labeled with anti-Nestin in the wt mouse. Frame 2 shows the cell bodies of the radial glial cells in the hyh mouse with a disrupted organization (asterisks). LV, Lateral Ventricle. Scale Bars: **(A)**, 200 μm; [Frames 1 and 2 from panel **(A)**], 90 μm; **(B)**, 120 μm; [Frames 1 and 2 from panel **(B)**], 40 μm; **(C)**, 200 μm; [Frames 1 and 2 from panel **(C)**], 20 μm.

The ventricular zone was then analyzed by immunostaining with anti-βIII-tubulin to check the distribution of the neuroblasts; anti-PCNA to identify proliferating cells in this area; and anti-Nestin to identify if radial glial cells were properly located (Figure 7 and Supplementary Figure 1). In the hyh mutant mouse, immunostaining with βIII-Tubulin in the region corresponding to the glial wedge cells showed invasion of cellular projections into the ventricular zone (Figure 7A). The immunolabeling with PCNA corroborated the alteration of the ventricular zone function in mutant animals (Figure 7B). Finally, the Nestin immunostaining revealed evident disorganization of this radial glial cell type in the hyh mutant mouse in the region where glial wedge cells should develop (Figure 7C). These results suggest that alteration of the radial glial cells in the ventricular zone corresponding to the glial wedge area could be responsible for the failure in the callosal axons independently of the ventricular dilatation.

### 3.7 Glial wedge signaling is altered in the hyh mutant mouse

**FIGURE 8:**
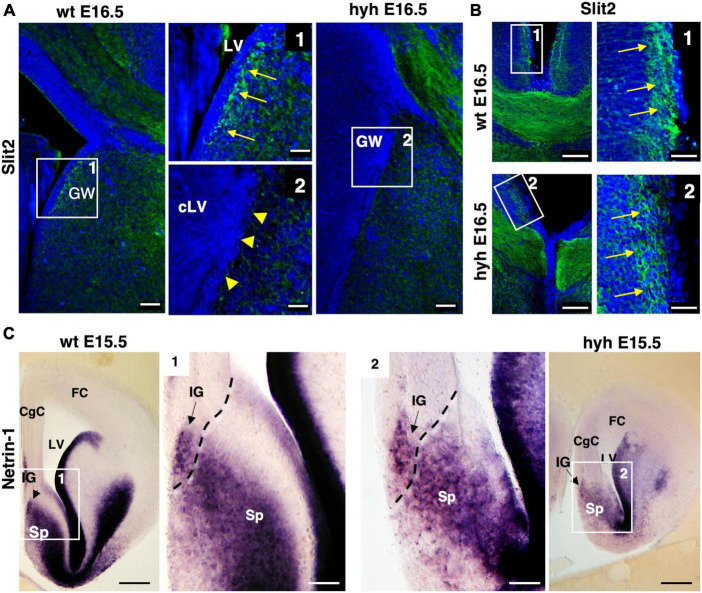
Slit2 and Netrin1 signaling. **(A)** Wild-type (wt) and hyh mutant mice brain frontal sections immunostained with anti-Slit2 antibody. Framed areas 1 (wt) and 2 (hyh) show the ventricular region where glial wedge cells should be. In the wt mouse (Frame 1), the Glial Wedge area (GW) is Slit2-positive (arrows). In the hyh mutant mouse (Frame 2), the ventricular region does not show positive immunolabeling (arrowheads). **(B)** Frontal brain sections of wt and hyh mutant mice immunostained with anti-Slit2 antibody. Framed areas 1 (wt) and 2 (hyh) show the immunolabeling in the indusium griseum glial cells (arrows). **(C)** *In situ* hybridization for Netrin1 in frontal sections of wt (left) and hyh mutant (right) mice brains. At E15.5, framed areas 1 (wt) and 2 (hyh) show the presence of Netrin1 mRNA in the indusium griseum glial cells and medial septum. The dashed line shows the approximated limit between pallial and subpallial areas. CgC, Cingulate Cortex; FC, Frontal Cortex; IG, Indusium Griseum; LV, Lateral Ventricle; cLV, collapsed Lateral Ventricle; Sp, Septum. Scale Bars: **(A)**, 50 μm; [Frames 1 and 2 in panel **(A)**], 15 μm; **(B)**, 100 μm; [Frames 1 and 2 in panel **(B)**], 40 μm; **(C)**, 200 μm [Frames 1 and 2 in panel **(C)**], 70 μm.

The Slit2 protein has been described to be secreted by indusium griseum glial cells and glial wedge cells for guiding the callosal pioneering axons (Shu et al., 2003c). This protein was examined in wt and hyh mice at E16.5. Results showed that the protein in the wt mouse was present in glial wedge cells (Figures 8A, B). However, in the hyh mouse, no Slit2 immunolabeling was detected where the glial wedge should be present, according to their selective disruption during the development (Figures 8A, B). Additionally, the expression of attractive signal Netrin1 involved in the attraction of callosal and pioneering axons from the indusium griseum glial cells and medial septum areas (Fothergill et al., 2014; Ahmed and Shinmyo, 2021) was studied and found with the same pattern in the wt and hyh mutant mice (Figure 8C).

### 3.8 Interhemispheric midline crossing of the pioneering axons

**FIGURE 9:**
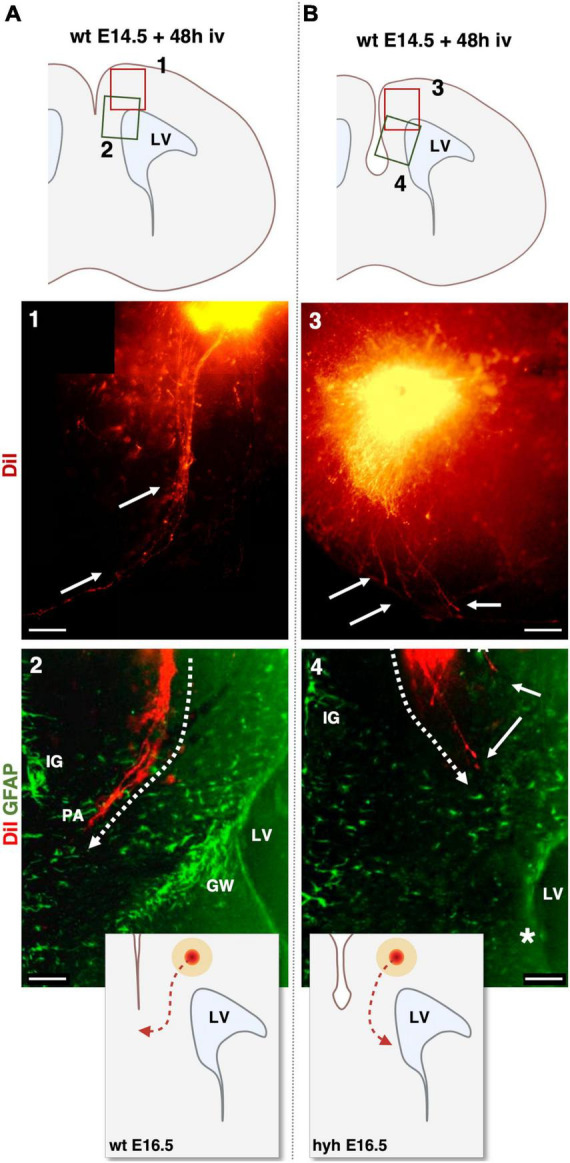
DiI-Tracing experiment in organotypic slice culture at E14.5 analyzed after 48 h *in vitro* (iv). DiI was applied to label the pioneering axons in organotypic mouse brain slices from wild-type [wt, **(A)**] and hyh mutant **(B)** mice. Top schemes show the location of detailed framed areas 1–4. Framed areas 1 and 3 show the DiI crystal position in the cingulate cortex and pioneering axons labeled fibers with DiI fluorescence (arrows). Framed areas 2 and 4 show the DiI fluorescence (red) overlapped with GFAP immunolabeling (green). Indusium Griseum (IG) glial cells and Glial Wedge (GW) cells are GFAP-positive in Frame 2. IG glial cells are GFAP positive in Frame 4. The GW cells are absent in the hyh mouse (asterisk) in Frame 4. Dashed lines in Frames 2 and 4 show the direction of elongation of the pioneering axons (PA). Schemes on the bottom show the differences in the pioneering axon routes. In the hyh mutant mouse, pioneering axons turn toward the lateral ventricle (LV). Scale Bars: 1 and 3, 100 μm; 2 and 4, 50 μm.

Knowing that glial wedge cells were necessary to guide the pioneering axons by E15.5 (Shu and Richards, 2001) and with evidence that at E15.5 glial wedge is altered or missing in the hyh mutant mouse, we decided to study the behavior of the pioneering axons at the corticoseptal boundary level. For this purpose, we analyzed the elongation of the pioneering axons in an organotypic culture system at E15.5. This experimental system also allowed us to exclude the possible contribution of environmental factors to the phenotype. DiI was applied into the cingulate cortex of brain slices from wt and hyh mice, obtained at E14.5 and E15.5 and maintained under organotypic culture conditions for 48 h *in vitro*. Results showed that in wt animals, pioneering axons are produced, and they started to cross the interhemispheric midline (Figures 9A, B). In contrast, in brain slices from hyh mice, it was observed that although those pioneering axons were elongating, at the level of the corticoseptal boundary, they were turning toward the ipsilateral lateral ventricle, thus failing to cross the midline (Figures 9A, B). The direction displayed by the pioneering axons toward the lateral ventricle in the absence of glial wedge cells supports the hypothesis that the lack of signaling molecules from the missing glial wedge cells is causing the dysgenesis of the corpus callosum.

## 4 Discussion

The present investigation has been performed with the hyh mutant mouse model presenting congenital hydrocephalus with an interhemispheric cyst and dysgenesis of the corpus callosum. During development, the hyh mouse presents a moderate communicating hydrocephalus (Jiménez et al., 2001). Over time, the hyh mouse develops an interhemispheric cyst covered with ependyma from the dorsal third ventricle (Pérez-Fígares et al., 1998). Hydrocephalus aggravation occurs during the first postnatal week when hydrocephalus in the hyh mouse became non-communicating (Wagner et al., 2003; Bátiz et al., 2006). Barkovich et al. (2001) have developed a classification of human cases with callosal dysgenesis of the corpus callosum. The present results show that the interhemispheric cyst in the hydrocephalic hyh mouse is not present at the developmental stages in which the corpus callosum is being generated. Moreover, in the collapsed lateral ventricles ventriculomegaly does not occur until postnatal ages in the hyh mouse. Collapse of the ventricles can be explained in base to the disruption of the neuroepithelium/differentiating ependyma in opposed ventricle walls, in the same way as happens in other parts of the lateral ventricle and cerebral aqueduct (Jiménez et al., 2001; Wagner et al., 2003). Therefore, the dysgenesis of the corpus callosum in the hyh mutant mouse model cannot be interpreted as a malformation due to the presence of an interhemispheric cyst or ventriculomegaly. It is plausible that the intrinsic defective program mechanism in the neuroepithelium leads to the genesis of the hydrocephalus and is also involved in the dysgenesis of the corpus callosum in the hyh mouse. This possibility must be investigated for its implications in similar human cases and the clinic.

Hyh mutants, due to the αSNAP mutation, present a defect in vesicle trafficking that seems to produce marked abnormalities in the location of F-Actin, α-Catenin, β-Catenin, E-Cadherin (Chae et al., 2004). These disturbances of junctional complexes are presumed to lead to an altered control of cell fate (Chae et al., 2004) and to a denudation of ventricular epithelium/radial glial cells in specific regions (Jiménez et al., 2001). The αSNAP defect produces an early differentiation of the neural progenitors by alteration of the decision between forming proliferative and postmitotic daughter cells, but without alteration in cell cycle kinetics (Chae et al., 2004). Early overproduction of neurons is done at the expense of progenitor cells (Chae et al., 2004). The denudation of the ventricular epithelium/radial glial cells progresses following a temporo-spatial pattern according to central nervous system development in the caudorostral and mediolateral axes (Jiménez et al., 2001). It irradiates from the pallial-subpallial limits in the telencephalon (Jiménez et al., 2001). The denudation of the neuroepithelial cells and the cell fate defect of neural progenitors could affect the normal development of the cells and structures involved in the corpus callosum formation. In this way, previous results have shown that the hyh mutant mice, even having a smaller cortex, present a normal forebrain patterning and laminar organization of the cortex by E14.5 (Chae et al., 2004). In the developed brain, the callosal projecting neurons are located in layers II-III and V (Fame et al., 2011). These cortical layers correspond to the cortical plate and intermediate zone in the embryonic cortical structure (Kolk and Rakic, 2022). These layers are mainly formed by glutamatergic neurons produced by the ventricular zone of the local pallium (Noctor et al., 2004; Rock et al., 2018) and with a small contribution of GABAergic interneurons generated from the subpallium, which reach the cerebral cortex via tangential migration (Ma et al., 2013; Hu et al., 2017; Puelles, 2017). In the hyh mouse, cell denudation does not affect the dorsal pallium until the postnatal stages (Jiménez et al., 2001). Therefore, most callosal projecting neurons should not be affected by denudation of the ventricular epithelium at E15.5 nor by the cell fate defect, which is corroborated by the presence of Probst bundles at E16.5 and by cortical structure analysis in the hyh mutant animals at E15.5 (Hematoxylin-Eosin, Calretinin, and Tbr1). The Tbr1 transcription factor has been described to be expressed in callosal neurons and pioneering axons and is needed for normal function (Koester and O’Leary, 1994). Tbr1 transcription factor has been detected in wt and hyh mice in the same cortical location, revealing the existence of the neurons responsible for generating the primary projecting axons of the callosal fibers (Bulfone et al., 1995; Hevner et al., 2001). In the mouse, GABAergic neurons migrate tangentially from the pallial-subpallial border through the intermedia and subventricular zones to the cortical plate at E12.5. Additionally, GABAergic interneurons from the subpallium migrate tangentially into the marginal and intermedia zones from E12.5 but do not enter the cortical plate until E14.5-E15.5 (Griveau et al., 2013; Laclef and Métin, 2018; Ahmed and Shinmyo, 2021). Neuroepithelium alteration in the subpallial area is not detected until E15.5 in the hyh mouse (Jiménez et al., 2001), thus suggesting that the presence of callosal projecting neurons should not be affected by the neuroepithelium defect of the hyh mutant mouse. Additionally, our results do not show any significant difference in the hyh mutant mouse for the Tag1 adhesion molecule, which mediates the migration of cortical interneurons from the ganglionic eminence (Denaxa et al., 2001).

In addition to the presence of the callosal projecting neurons, the proper balance between attractive and repellent cues in the callosal axon growth cone is fundamental. Different molecules and receptors have been reported as a determinant for the appropriate behavior of the growth cone, such as Nogo receptors (Yoo et al., 2017), Robo receptors, Slit molecules (Unni et al., 2012 w12), or Tag1 (Wolman et al., 2008). The protein Tag1 plays a critical role in the initial phase of the growth of the neurites (Furley et al., 1990). Tag1 is a member of the immunoglobulin superfamily that, in addition to axon outgrowth, also plays a role in migration and fasciculation during development (Karagogeos, 2003). Tag1 has been described as present in the fibers forming the corpus callosum (Fujimori et al., 2000; Savvaki et al., 2008). Interestingly, in both wt and hyh mice, the Tag1 molecule is expressed in the cortical layer where callosal neurons and pioneering axons reside. In the same way, tracing axonal growth with DiI at P1 showed that the axons from callosal neurons could grow toward the midline, indicating no presumable defects in the ability of the neurons to extend their projections and possible failure in the structures implicated in their guidance.

Axonal guidance during corpus callosum development is possible by cingulate pioneering axons, subcallosal sling, glial wedge, indusium griseum glial cells (Richards et al., 2004). The cingulate pioneering axons guide the callosal axons during the first stage of the midline crossing (Rash and Richards, 2001; Shu et al., 2003b). By using GAP-43 immunofluorescence, we have found that pioneering axons are present in the cingulate cortex at E15.5 in both hydrocephalic hyh and wt mice, indicating the neuroepithelial cell alteration existing in the hyh mutant mice is not affecting the pioneering axons. The neuron sling does not participate in the guidance of pioneering axons (Shu et al., 2003a) but could also be involved in the guidance of callosal axons. However, the neuron sling is present in the hyh mouse when the corpus callosum develops, indicating that the responsible defect should be in one of the glial cell populations.

The glial wedge cells are considered part of the radial glial cells scaffold of the cerebral cortex, expressing some markers such as GFAP or Nestin (Shu et al., 2003b), which are developed directly from the neuroepithelium. Neuroepithelial cells and radial glia cells include mixture of subpopulations with differential cell markers and a variable extent of fate restriction (Kriegstein and Alvarez-Buylla, 2009; Taverna et al., 2014; Garcia-Calero and Puelles, 2020). Hyh mutant mice present low levels of protein αSNAP that can be detected after the transition from neuroepithelial cell into radial glial cells (Chae et al., 2004). These alteration leads to abnormal cell junctions in the cell membrane of ventricular epithelium and seems to cause detachment of affected cells (Chae et al., 2004; Rodríguez et al., 2012) in a well-defined program (Jiménez et al., 2001). Each subpopulation of radial glial cell could be also analyzed as radial histogenetic unit that share the same molecular profile (Garcia-Calero and Puelles, 2020) and that differentiates at different moments during brain development depending on the specific location (Tramontin et al., 2003; Taverna et al., 2014; Garcia-Calero and Puelles, 2020). Consequently, the αSNAP mutation starts to cause cell adhesion defects in the radial glial cells as they differentiate and mature, and the detachment of affected radial glial cells is detected as patches that extend over time. In this regard, the cells of the glial wedge are located in the limit pallium-subpallium at the septal level (Puelles et al., 2000; Puelles, 2017), differentiate earlier than the neighbor neuroepithelium and express GFAP before other regions of the dorsal telencephalon (Shu and Richards, 2001; Shu et al., 2003b). In the hyh mutant mouse, this subpopulation of glial cells is specifically affected at E15.5, a critical moment for corpus callosum development. Both glial wedge cells and indusium griseum cells are formed from radial glia at the cortico-septal boundary (Shu et al., 2003b; Smith et al., 2006). At E14.5, radial glial in such region detach from the ventricular zone, migrate to the pial surface and differentiate into indusium griseum glial cells (Smith et al., 2006); special astrocytes placed underneath the medial pial membrane (Shu et al., 2003b). Most indusium griseum glial cells are born at E14.5 and E15.5, with only a few generated at E17 (Shu et al., 2003b). Neuroepithelium/radial glial alteration in the region corresponding to the glial wedge (corticoseptal boundary) is detected from E15.5 (Jiménez et al., 2001). Therefore, when pioneering axons must cross the midline, the indusium griseum glial cells generated between E14-E15.5 should be present. We have proved their presence with GFAP at E17.5. Finally, with GFAP labeling we have also detected the presence of midline zipper glia (MZG) under the Probst bundles indicating that the absence of the glial wedge population is specific.

Midline zipper glia mature between E14 and E17 and regulate interhemispheric remodeling through multiple molecular mechanisms, including Draxin-signaling (Gobius et al., 2016; Morcom et al., 2021). A lack of interhemispheric fusion is a major cause of corpus callosum dysgenesis (Gobius et al., 2016). In the hyh mouse, MZG still forms, but in some animals, interhemispheric fusion failed to occur fully by E17, which may contribute to the phenotype. When the pioneering axons are elongating to cross the midline, E15.5, no defects at the interhemispheric fusion are detected. A defect in the MZG could affect the posterior phases of the callosal projection axons crossing. Although MZG developed, their maturation could be delayed or affected by the defects in glial wedge development or directly through genetic mutations in the hyh mouse.

The indusium griseum cortical area is specified between E13.5 and E15.5 from the corticoseptal boundary area that delimits pallial and subpallial telencephalic regions. Molecular subdivisions postulated in the tetrapartite pallial model of Puelles et al. (2000) are defined through the expression of different genes such as Pax6, Emx1, Dlx2, Nkk2.1, and Tbr1. Tbr1 and Emx1 are differentially expressed in the pallium, and Dlx2 and Nkk2.1 in the subpallium (Puelles et al., 2000; Puelles, 2017). Tbr1 is especially interesting as it defines the limit pallium-subpallium at the corticoseptal boundary. Tbr1 is expressed in the pallial region, including the indusium griseum cortical area at E15.5. Our analysis of Tbr1 shows no significant differences in the cingular cortex of the hyh mouse at E14.5, a critical time in which the indusium griseum glial cells are developing. Even if the hyh mutant mouse brain has a reduced size, no alteration in the telencephalic embryonic regionalization that could be related to the alteration of the corpus callosum formation has been detected.

The populations participating in the guidance of the callosal axons during the corpus callosum formation exert their function through secreted molecules, including extracellular matrix components and different soluble molecules like Slits, Netrins, Wnt family, and FGF and their receptors (Lindwall et al., 2007). The glial wedge and indusium griseum glial cells are known sources of chemorepellent molecules such as Slit2 (Shu et al., 2003c) and attractive cues like Draxin-Netrin1 system (Fothergill et al., 2014; Ahmed and Shinmyo, 2021). Both populations create a pathway for the callosal axons to cross the midline. If repellant and attractive signaling fails from both cell populations in the hyh mutant mouse, pioneering axons should be projecting in all directions. However, in our study, Slit2 and Netrin1 signal are present in the mutant animals. Additionally, the organotypic culture at E15.5 has shown that the pioneering axons in the hyh mutant animals elongate toward the septum (probably attracted by Netrin1) but turned toward the lateral ventricle in the region corresponding to the missing glial wedge cells. This defective direction can be explained by the presence of the repellant cues effect from the indusium griseum cells and their absence from the glial wedge cells.

For the normal functioning and development of the brain, homeostasis must be maintained (Castells et al., 2012; Rasmussen et al., 2022). Defects in neuroepithelium development can affect brain homeostasis differently, such as the barrier between the cerebrospinal fluid (CSF) and the brain parenchyma (Jiménez et al., 2014; Duy et al., 2023). Then, the correct axonal guidance microenvironment is modified, and the different soluble molecules acting as repellent cues may not function correctly (Jiménez et al., 2014; Duy et al., 2023). Additionally, altering the ventricular zone may alter the normal CSF circulation (Jiménez et al., 2014) and produce an accumulation of toxins in the parenchyma (Owen-Lynch et al., 2003; Lubinsky, 2022). CSF composition influences normal brain development (Mashayekhi and Salehi, 2006; Castells et al., 2012; Rasmussen et al., 2022). In the case of hydrocephalic H-Tx rats, it has been shown that the cerebrospinal fluid negatively influences cortical development (Mashayekhi et al., 2002; Owen-Lynch et al., 2003). Therefore, the alteration of neuroepithelium could affect not only the populations involved in corpus callosum development but also the signaling of these populations indirectly by disrupting the local microenvironment. We cannot discard the possibility that other signaling molecules from cell populations involved in the corpus callosum development and still present in the hyh mutant mice could be altered in the disrupted ventricular areas.

In conclusion, the present investigation shows a direct link between neuroepithelium/radial glial cell damage, congenital hydrocephalus and the dysgenesis of the corpus callosum. This link is, additionally, independent of the ventriculomegaly. Some studies have previously reported that congenital hydrocephalus may be the result of developmental neuroepithelial disorders associated with impaired cellular development and signaling (Domínguez-Pinos et al., 2005; Jiménez et al., 2014; Guerra et al., 2015; Hochstetler et al., 2022). Considering the present results, in cases of congenital hydrocephalus concurring with neuroepithelial cell alteration, an early analysis of callosal failures is needed to predict the prognosis and decide the appropriate therapy.

## Data availability statement

The raw data supporting the conclusions of this article will be made available by the authors, without undue reservation.

## Ethics statement

The animal study was approved by the Institutional Animal Care and Use Committee of the University of Malaga (CEUMA) and the Regional Government Council (Junta de Andalucía, Spain). The study was conducted in accordance with the local legislation and institutional requirements.

## Author contributions

L-MR-P: Conceptualization, Data curation, Formal analysis, Investigation, Methodology, Validation, Visualization, Writing—original draft, Writing—review and editing. JL-d-S-S: Investigation, Software, Visualization, Writing—review and editing, Data curation. ID: Data curation, Investigation, Visualization, Writing—review and editing, Validation. AS: Data curation, Investigation, Visualization, Writing—review and editing. RR-B: Investigation, Writing—review and editing, Methodology. AJ: Conceptualization, Funding acquisition, Investigation, Methodology, Supervision, Visualization, Writing—original draft, Writing—review and editing, Data curation, Formal analysis, Project administration, Validation. PP-G: Conceptualization, Data curation, Formal analysis, Funding acquisition, Investigation, Methodology, Supervision, Validation, Visualization, Writing—original draft, Writing—review and editing, Project administration.

## References

[B1] Achiron R. Achiron A. (2001). Development of the human fetal corpus callosum: a high-resolution, cross-sectional sonographic study. *Ultrasound Obstet. Gynecol.* 18 343–347. 10.1046/J.0960-7692.2001.00512.X 11778993

[B2] Adle-Biassette H. Saugier-Veber P. Fallet-Bianco C. Delezoide A. L. Razavi F. Drouot N. (2013). Neuropathological review of 138 cases genetically tested for X-linked hydrocephalus: evidence for closely related clinical entities of unknown molecular bases. *Acta Neuropathol.* 126 427–442. 10.1007/S00401-013-1146-1 23820807

[B3] Ahmed G. Shinmyo Y. (2021). Multiple functions of Draxin/Netrin-1 signaling in the development of neural circuits in the spinal cord and the brain. *Front. Neuroanat.* 15:766911. 10.3389/fnana.2021.766911 34899198 PMC8655782

[B4] Ahmed R. R. Medhat A. M. Hamdy G. M. Effat L. K. E. Abdel-Hamid M. S. Abdel-Salam G. M. H. (2023). X-linked hydrocephalus with new L1CAM pathogenic variants: review of the most prevalent molecular and phenotypic features. *Mol. Syndromol.* 14 283–292. 10.1159/000529545 37766829 PMC10521243

[B5] Barkovich A. J. Norman D. (1988). Anomalies of the corpus callosum: correlation with further anomalies of the brain. *Am. J. Roentgenol.* 151 171–179. 10.2214/AJR.151.1.171 3259802

[B6] Barkovich A. J. Simon E. M. Walsh C. A. (2001). Callosal agenesis with cyst: a better understanding and new classification. *Neurology* 56 220–227. 10.1212/WNL.56.2.220 11160959

[B7] Barresi M. J. F. Hutson L. D. Chien C.-B. Karlstrom R. O. (2005). Hedgehog regulated Slit expression determines commissure and glial cell position in the zebrafish forebrain. *Development* 132 3643–3656. 10.1242/dev.01929 16033800

[B8] Bátiz L. F. Páez P. Jiménez A. J. Rodríguez S. Wagner C. Pérez-Fígares J. M. (2006). Heterogeneous expression of hydrocephalic phenotype in the hyh mice carrying a point mutation in alpha-SNAP. *Neurobiol. Dis.* 23 152–168. 10.1016/J.NBD.2006.02.009 16697210

[B9] Bátiz L. F. Roales-Buján R. Rodríguez-Pérez L. M. Matas I. M. Páez P. Roque M. (2009). A simple PCR-based genotyping method for M105I mutation of alpha-SNAP enhances the study of early pathological changes in hyh phenotype. *Mol. Cell Probes* 23 281–290. 10.1016/j.mcp.2009.07.002 19615440

[B10] Booth G. E. Kinrade E. F. V. Hidalgo A. (2000). Glia maintain follower neuron survival during Drosophila CNS development. *Development* 127 237–244. 10.1242/DEV.127.2.237 10603342

[B11] Bronson R. T. Lane P. W. (1990). Hydrocephalus with hop gait (hyh): a new mutation on chromosome 7 in the mouse. *Brain Res. Dev. Brain Res.* 54 131–136. 10.1016/0165-3806(90)90073-8 2364541

[B12] Bulfone A. Smiga S. M. Shimamura K. Peterson A. Puelles L. Rubenstein J. L. (1995). T-brain-1: a homolog of Brachyury whose expression defines molecularly distinct domains within the cerebral cortex. *Neuron* 15 63–78. 10.1016/0896-6273(95)90065-9 7619531

[B13] Castells A. Parvas M. Bueno D. (2012). Homeostasis of cerebrospinal fluid has a role in early brain development. *Neuroreport* 23 917–921. 10.1097/WNR.0B013E3283582067 22922657

[B14] Chae T. H. Kim S. Marz K. E. Hanson P. I. Walsh C. A. (2004). The hyh mutation uncovers roles for alpha Snap in apical protein localization and control of neural cell fate. *Nat. Genet.* 36 264–270. 10.1038/ng1302 14758363

[B15] Clary D. O. Griff I. C. Rothman J. E. (1990). SNAPs, a family of NSF attachment proteins involved in intracellular membrane fusion in animals and yeast. *Cell* 61 709–721. 10.1016/0092-8674(90)90482-T 2111733

[B16] Crespo I. Pignatelli J. Kinare V. Méndez-Gómez H. R. Esgleas M. Román M. J. (2022). Tbr1 Misexpression alters neuronal development in the cerebral cortex. *Mol. Neurobiol.* 59 5750–5765. 10.1007/S12035-022-02936-X 35781633 PMC9395452

[B17] de Diego I. Kyriakopoulou K. Karagogeos D. Wassef M. (2002). Multiple influences on the migration of precerebellar neurons in the caudal medulla. *Development* 129 297–306. 10.1242/DEV.129.2.297 11807023

[B18] Demyanenko G. P. Tsai A. Y. Maness P. F. (1999). Abnormalities in neuronal process extension, hippocampal development, and the ventricular system of L1 knockout mice. *J. Neurosci.* 19 4907–4920. 10.1523/JNEUROSCI.19-12-04907.1999 10366625 PMC6782672

[B19] Denaxa M. Chan C. H. Schachner M. Parnavelas J. G. Karagogeos D. (2001). The adhesion molecule TAG-1 mediates the migration of cortical interneurons from the ganglionic eminence along the corticofugal fiber system. *Development* 128 4635–4644. 10.1242/DEV.128.22.4635 11714688

[B20] Diogo M. C. Glatter S. Prayer D. Gruber G. M. Bettelheim D. Weber M. (2021). Improved neurodevelopmental prognostication in isolated corpus callosal agenesis: fetal magnetic resonance imaging-based scoring system. *Ultrasound Obstet. Gynecol.* 58 34–41. 10.1002/UOG.22102 32484578 PMC8362015

[B21] Domínguez-Pinos M. D. Páez P. Jiménez A. J. Weil B. Arráez M. A. Pérez-Fígares J. M. (2005). Ependymal denudation and alterations of the subventricular zone occur in human fetuses with a moderate communicating hydrocephalus. *J. Neuropathol. Exp. Neurol.* 64 595–604. 10.1097/01.JNEN.0000171648.86718.BB 16042311

[B22] Duy P. Q. Rakic P. Alper S. L. Robert S. M. Kundishora A. J. Butler W. E. (2023). A neural stem cell paradigm of pediatric hydrocephalus. *Cereb. Cortex* 33 4262–4279. 10.1093/CERCOR/BHAC341 36097331 PMC10110448

[B23] Edwards T. J. Sherr E. H. Barkovich A. J. Richards L. J. (2014). Clinical, genetic and imaging findings identify new causes for corpus callosum development syndromes. *Brain* 137 1579–1613. 10.1093/BRAIN/AWT358 24477430 PMC4032094

[B24] Fame R. M. MacDonald J. L. Macklis J. D. (2011). Development, specification, and diversity of callosal projection neurons. *Trends Neurosci.* 34 41–50. 10.1016/J.TINS.2010.10.002 21129791 PMC3053014

[B25] Ferland R. J. Batiz L. F. Neal J. Lian G. Bundock E. Lu J. (2009). Disruption of neural progenitors along the ventricular and subventricular zones in periventricular heterotopia. *Hum. Mol. Genet.* 18 497–516. 10.1093/HMG/DDN377 18996916 PMC2722192

[B26] Fothergill T. Donahoo A. L. S. Douglass A. Zalucki O. Yuan J. Shu T. (2014). Netrin-DCC signaling regulates corpus callosum formation through attraction of pioneering axons and by modulating Slit2-mediated repulsion. *Cereb. Cortex* 24 1138–1151. 10.1093/CERCOR/BHS395 23302812

[B27] Fujimori K. E. Takeuchi K. Yazaki T. Uyemura K. Nojyo Y. Tamamki N. (2000). Expression of L1 and TAG-1 in the corticospinal, callosal, and hippocampal commissural neurons in the developing rat telencephalon as revealed by retrograde and in situ hybridization double labeling. *J. Comp. Neurol.* 417 275–288. 10.1002/(sici)1096-9861(20000214)417:3<275::aid-cne2>3.0.co;2-7 10683603

[B28] Furley A. J. Morton S. B. Manalo D. Karagogeos D. Dodd J. Jessell T. M. (1990). The axonal glycoprotein TAG-1 is an immunoglobulin superfamily member with neurite outgrowth-promoting activity. *Cell* 61 157–170. 10.1016/0092-8674(90)90223-2 2317872

[B29] Garcia-Calero E. Puelles L. (2020). Histogenetic radial models as aids to understanding complex brain structures: the amygdalar radial model as a recent example. *Front. Neuroanat.* 14:590011. 10.3389/FNANA.2020.590011 33240050 PMC7683391

[B30] Gobius I. Morcom L. Suárez R. Bunt J. Bukshpun P. Reardon W. (2016). Astroglial-mediated remodeling of the interhemispheric midline is required for the formation of the corpus callosum. *Cell Rep.* 17 735–747. 10.1016/J.CELREP.2016.09.033 27732850 PMC5094913

[B31] González-Gómez M. Meyer G. (2014). Dynamic expression of calretinin in embryonic and early fetal human cortex. *Front. Neuroanat.* 8:41. 10.3389/FNANA.2014.00041 24917793 PMC4042362

[B32] Griveau A. Borello U. Pierani A. (2013). “Neuronal migration and brain patterning,” in *Cellular Migration and Formation of Neuronal Connections: Comprehensive Developmental Neuroscience*, eds. Rubenstein J. L. R. Rakic P. (Cambridge, MA: Academic Press).

[B33] Guerra M. M. Henzi R. Ortloff A. Lichtin N. Vío K. Jiménez A. J. (2015). Cell junction pathology of neural stem cells is associated with ventricular zone disruption, hydrocephalus, and abnormal neurogenesis. *J. Neuropathol. Exp. Neurol.* 74 653–671. 10.1097/NEN.0000000000000203 26079447

[B34] Hernandez N. E. Lu V. M. Altman N. Ragheb J. Niazi T. N. Wang S. (2022). Incidence, follow-up, and postnatal clinical progress of children with central nervous system anomalies on fetal MRI. *J. Neurosurg. Pediatr.* 30 160–168. 10.3171/2022.4.PEDS2269 35901770

[B35] Hevner R. F. Shi L. Justice N. Hsueh Y. P. Sheng M. Smiga S. (2001). Tbr1 regulates differentiation of the preplate and layer 6. *Neuron* 29 353–366. 10.1016/S0896-6273(01)00211-2 11239428

[B36] Hidalgo A. Booth G. E. (2000). Glia dictate pioneer axon trajectories in the Drosophila embryonic CNS. *Development* 127 393–402. 10.1242/DEV.127.2.393 10603355

[B37] Hochstetler A. Raskin J. Blazer-Yost B. L. (2022). Hydrocephalus: historical analysis and considerations for treatment. *Eur. J. Med. Res.* 27:168. 10.1186/s40001-022-00798-6 PMC9434947 36050779

[B38] Hofman J. Hutny M. Sztuba K. Paprocka J. (2020). Corpus callosum agenesis: an insight into the etiology and spectrum of symptoms. *Brain Sci.* 10:625. 10.3390/brainsci10090625 PMC7565833 32916978

[B39] Hong H.-K. Chakravarti A. Takahashi J. S. (2004). The gene for soluble N-ethylmaleimide sensitive factor attachment protein alpha is mutated in hydrocephaly with hop gait (hyh) mice. *Proc. Natl. Acad. Sci. U S A.* 101 1748–1753. 10.1073/pnas.0308268100 14755058 PMC341847

[B40] Hu J. S. Vogt D. Sandberg M. Rubenstein J. L. (2017). Cortical interneuron development: a tale of time and space. *Development* 144 3867–3878. 10.1242/DEV.132852 29089360 PMC5702067

[B41] Islam S. M. Shinmyo Y. Okafuji T. Su Y. Naser I. Bin (2009). Draxin, a repulsive guidance protein for spinal cord and forebrain commissures. *Science* 323 388–393. 10.1126/science.1165187 19150847

[B42] Jiménez A. J. Domínguez-Pinos M. D. Guerra M. M. Fernández-Llebrez P. Pérez-Fígares J. M. (2014). Structure and function of the ependymal barrier and diseases associated with ependyma disruption. *Tissue Barriers* 2: e28426. 25045600 10.4161/tisb.28426 PMC4091052

[B43] Jiménez A. J. Tomé M. Páez P. Wagner C. Rodríguez S. Fernández-Llebrez P. (2001). A programmed ependymal denudation precedes congenital hydrocephalus in the hyh mutant mouse. *J. Neuropathol. Exp. Neurol.* 60 1105–1119. 10.1093/JNEN/60.11.1105 11706940

[B44] Jin S. C. Dong W. Kundishora A. J. Panchagnula S. Moreno-De-Luca A. Furey C. G. (2020). Exome sequencing implicates genetic disruption of prenatal neuro-gliogenesis in sporadic congenital hydrocephalus. *Nat. Med.* 26 1754–1765. 10.1038/S41591-020-1090-2 33077954 PMC7871900

[B45] Jovanov-Milošević N. Čuljat M. Kostović I. (2009). Growth of the human corpus callosum: modular and laminar morphogenetic zones. *Front. Neuroanat.* 3:6. 10.3389/neuro.05.006.2009 19562029 PMC2697006

[B46] Karagogeos D. (2003). Neural GPI-anchored cell adhesion molecules. *Front. Biosci.* 8:s1304-20. 10.2741/1214 12957835

[B47] Kastriti M. E. Stratigi A. Mariatos D. Theodosiou M. Savvaki M. Kavkova M. (2019). Ablation of CNTN2+ pyramidal neurons during development results in defects in neocortical size and axonal tract formation. *Front. Cell Neurosci.* 13:454. 10.3389/fncel.2019.00454 31749685 PMC6844266

[B48] Keeble T. R. Halford M. M. Seaman C. Kee N. Macheda M. Anderson R. B. (2006). The Wnt receptor Ryk is required for Wnt5a-mediated axon guidance on the contralateral side of the corpus callosum. *J. Neurosci.* 26 5840–5848. 10.1523/JNEUROSCI.1175-06.2006 16723543 PMC6675257

[B49] Kier E. Truwit C. (1997). The lamina rostralis: modification of concepts concerning the anatomy, embryology, and MR appearance of the rostrum of the corpus callosum. *Am. J. Neuroradiol.* 18 715–722. 9127036 PMC8338510

[B50] Koester S. E. O’Leary D. D. M. (1994). Axons of early generated neurons in cingulate cortex pioneer the corpus callosum. *J. Neurosci.* 14 6608–6620. 10.1523/JNEUROSCI.14-11-06608.1994 7965064 PMC6577278

[B51] Kolk S. M. Rakic P. (2022). Development of prefrontal cortex. *Neuropsychopharmacology* 47 41–57. 10.1038/S41386-021-01137-9 34645980 PMC8511863

[B52] Kriegstein A. Alvarez-Buylla A. (2009). The glial nature of embryonic and adult neural stem cells. *Annu. Rev. Neurosci.* 32 149–184. 10.1146/ANNUREV.NEURO.051508.135600 19555289 PMC3086722

[B53] Krupa K. Bekiesinska-Figatowska M. (2013). Congenital and acquired abnormalities of the corpus callosum: a pictorial essay. *Biomed. Res. Int.* 2013:265619. 10.1155/2013/265619 PMC3763572 24027754

[B54] Laclef C. Anselme I. Besse L. Catala M. Palmyre A. Baas D. (2015). The role of primary cilia in corpus callosum formation is mediated by production of the Gli3 repressor. *Hum. Mol. Genet.* 24 4997–5014. 10.1093/HMG/DDV221 26071364

[B55] Laclef C. Métin C. (2018). Conserved rules in embryonic development of cortical interneurons. *Semin. Cell Dev. Biol.* 76 86–100. 10.1016/J.SEMCDB.2017.09.017 28918121

[B56] Lent R. Uziel D. Baudrimont M. Fallet C. (2005). Cellular and molecular tunnels surrounding the forebrain commissures of human fetuses. *J. Comp. Neurol.* 483 375–382. 10.1002/CNE.20427 15700272

[B57] Lindwall C. Fothergill T. Richards L. J. (2007). Commissure formation in the mammalian forebrain. *Curr. Opin. Neurobiol.* 17 3–14. 17275286 10.1016/j.conb.2007.01.008

[B58] Lubinsky M. (2022). Hypothesis: by-products of vascular disruption carried in the CSF affect prenatal brain development. *Birth Defects Res.* 114 847–854. 10.1002/BDR2.2064 35775635

[B59] Ma T. Wang C. Wang L. Zhou X. Tian M. Zhang Q. (2013). Subcortical origins of human and monkey neocortical interneurons. *Nat. Neurosci.* 16 1588–1597. 10.1038/NN.3536 24097041

[B60] Malinger G. Zakut H. (1993). The corpus callosum: normal fetal development as shown by transvaginal sonography. *Am. J. Roentgenol.* 161 1041–1043. 10.2214/AJR.161.5.8273605 8273605

[B61] Mashayekhi F. Draper C. E. Bannister C. M. Pourghasem M. Owen-Lynch P. J. Miyan J. A. (2002). Deficient cortical development in the hydrocephalic Texas (H-Tx) rat: a role for CSF. *Brain* 125 1859–1874. 10.1093/brain/awf182 12135976

[B62] Mashayekhi F. Salehi Z. (2006). The importance of cerebrospinal fluid on neural cell proliferation in developing chick cerebral cortex. *Eur. J. Neurol.* 13 266–272. 10.1111/J.1468-1331.2006.01208.X 16618344

[B63] Masmejan S. Blaser S. Keunen J. Seaward G. Windrim R. Kelly E. (2020). Natural history of ventriculomegaly in fetal agenesis of the corpus callosum. *J. Ultrasound Med.* 39 483–488. 10.1002/jum.15124 31502300

[B64] Morcom L. Edwards T. J. Rider E. Jones-Davis D. Lim J. W. C. Chen K. S. (2021). DRAXIN regulates interhemispheric fissure remodelling to influence the extent of corpus callosum formation. *Elife* 10:e61618. 10.7554/eLife.61618 33945466 PMC8137145

[B65] Morris J. K. Wellesley D. G. Barisic I. Addor M. C. Bergman J. E. H. Braz P. (2019). Epidemiology of congenital cerebral anomalies in Europe: a multicentre, population-based EUROCAT study. *Arch. Dis. Child* 104 1181–1187. 10.1136/ARCHDISCHILD-2018-316733 31243007

[B66] Nishikimi M. Oishi K. Nakajima K. (2013). Axon guidance mechanisms for establishment of callosal connections. *Neural Plast* 2013:149060. 10.1155/2013/149060 PMC3595665 23533817

[B67] Noctor S. C. Martinez-Cerdeño V. Ivic L. Kriegstein A. R. (2004). Cortical neurons arise in symmetric and asymmetric division zones and migrate through specific phases. *Nat. Neurosci.* 7 136–144. 10.1038/NN1172 14703572

[B68] Oestreicher A. B. De Graan P. N. E. Gispen W. H. Verhaagen J. Schrama L. H. (1997). B-50, the growth associated protein-43: modulation of cell morphology and communication in the nervous system. *Prog. Neurobiol.* 53 627–686. 10.1016/S0301-0082(97)00043-9 9447616

[B69] Owen-Lynch P. J. Draper C. E. Mashayekhi F. Bannister C. M. Miyan J. A. (2003). Defective cell cycle control underlies abnormal cortical development in the hydrocephalic Texas rat. *Brain* 126 623–631. 10.1093/BRAIN/AWG058 12566283

[B70] Paez P. Bátiz L.-F. Roales-Buján R. Rodríguez-Pérez L.-M. Rodríguez S. Jiménez A. J. (2007). Patterned neuropathologic events occurring in hyh congenital hydrocephalic mutant mice. *J. Neuropathol. Exp. Neurol.* 66 1082–1092. 10.1097/nen.0b013e31815c1952 18090917

[B71] Pânzaru M. C. Popa S. Lupu A. Gavrilovici C. Lupu V. V. Gorduza E. V. (2022). Genetic heterogeneity in corpus callosum agenesis. *Front. Genet.* 13:958570. 10.3389/FGENE.2022.958570 36246626 PMC9562966

[B72] Paul L. K. Brown W. S. Adolphs R. Tyszka J. M. Richards L. J. Mukherjee P. (2007). Agenesis of the corpus callosum: genetic, developmental and functional aspects of connectivity. *Nat. Rev. Neurosci.* 8 287–299. 10.1038/NRN2107 17375041

[B73] Pérez-Fígares J. M. Jiménez A. J. Pérez-Martín M. Fernández-Llebrez P. Cifuentes M. Riera P. (1998). Spontaneous congenital hydrocephalus in the mutant mouse hyh. changes in the ventricular system and the subcommissural organ. *J. Neuropathol. Exp. Neurol.* 57 188–202. 10.1097/00005072-199802000-00010 9600211

[B74] Puelles L. (2017). Comments on the updated tetrapartite pallium model in the mouse and chick, featuring a homologous claustro-insular complex. *Brain Behav. Evol.* 90 171–189. 10.1159/000479782 28988246

[B75] Puelles L. Kuwana E. Puelles E. Bulfone A. Shimamura K. Keleher J. (2000). Pallial and subpallial derivatives in the embryonic chick and mouse telencephalon, traced by the expression of the genes Dlx-2, Emx-1, Nkx-2.1, Pax-6, and Tbr-1. *J. Comp. Neurol.* 424 409–438. 10906711 10.1002/1096-9861(20000828)424:3<409::aid-cne3>3.0.co;2-7

[B76] Püschel A. W. O’Connor V. Betz H. (1994). The N-ethylmaleimide-sensitive fusion protein (NSF) is preferentially expressed in the nervous system. *FEBS Lett.* 347 55–58. 10.1016/0014-5793(94)00505-2 8013662

[B77] Rakic P. Yakovlev P. I. (1968). Development of the corpus callosum and cavum septi in man. *J. Comp. Neurol.* 132 45–72. 10.1002/CNE.901320103 5293999

[B78] Rash B. G. Richards L. J. (2001). A role for cingulate pioneering axons in the development of the corpus callosum. *J. Comp. Neurol.* 434 147–157. 10.1002/CNE.1170 11331522

[B79] Rasmussen M. K. Mestre H. Nedergaard M. (2022). Fluid transport in the brain. *Physiol. Rev.* 102 1025–1151. 10.1152/PHYSREV.00031.2020 33949874 PMC8897154

[B80] Raybaud C. (2010). The corpus callosum, the other great forebrain commissures, and the septum pellucidum: anatomy, development, and malformation. *Neuroradiology* 52 447–477. 10.1007/S00234-010-0696-3 20422408

[B81] Ren T. Anderson A. Shen W. Bin, Huang H. Plachez C. (2006). Imaging, anatomical, and molecular analysis of callosal formation in the developing human fetal brain. *Anat. Rec. A Discov. Mol. Cell. Evol. Biol.* 288 191–204. 10.1002/AR.A.20282 16411247

[B82] Richards L. J. Plachez C. Ren T. (2004). Mechanisms regulating the development of the corpus callosum and its agenesis in mouse and human. *Clin. Genet.* 66 276–289. 10.1111/J.1399-0004.2004.00354.X 15355427

[B83] Rock C. Zurita H. Lebby S. Wilson C. J. Apicella A. J. (2018). Cortical circuits of callosal GABAergic neurons. *Cereb. Cortex* 28 1154–1167. 10.1093/CERCOR/BHX025 28174907

[B84] Rodríguez E. M. Guerra M. M. Vío K. González C. Ortloff A. Bátiz L. F. (2012). A cell junction pathology of neural stem cells leads to abnormal neurogenesis and hydrocephalus. *Biol. Res.* 45 231–241. 10.4067/S0716-97602012000300005 23283433

[B85] Rubenstein J. L. R. Shimamura K. Martinez S. Puelles L. (1998). Regionalization of the prosencephalic neural plate. *Annu. Rev. Neurosci.* 21 445–477. 10.1146/ANNUREV.NEURO.21.1.445 9530503

[B86] Savvaki M. Panagiotaropoulos T. Stamatakis A. Sargiannidou I. Karatzioula P. Watanabe K. (2008). Impairment of learning and memory in TAG-1 deficient mice associated with shorter CNS internodes and disrupted juxtaparanodes. *Mol. Cell. Neurosci.* 39 478–490. 10.1016/j.mcn.2008.07.025 18760366

[B87] Seki T. Arai Y. (1993). Distribution and possible roles of the highly polysialylated neural cell adhesion molecule (NCAM-H) in the developing and adult central nervous system. *Neurosci. Res.* 17 265–290. 10.1016/0168-0102(93)90111-3 8264989

[B88] Shu T. Li Y. Keller A. Richards L. J. (2003a). The glial sling is a migratory population of developing neurons. *Development* 130 2929–2937. 10.1242/DEV.00514 12756176 PMC2810520

[B89] Shu T. Puche A. C. Richards L. J. (2003b). Development of midline glial populations at the corticoseptal boundary. *J. Neurobiol.* 57 81–94. 10.1002/NEU.10252 12973830

[B90] Shu T. Sundaresan V. McCarthy M. M. Richards L. J. (2003c). Slit2 guides both precrossing and postcrossing callosal axons at the midline in vivo. *J. Neurosci.* 23 8176–8184. 10.1523/JNEUROSCI.23-22-08176.2003 12954881 PMC6740498

[B91] Shu T. Richards L. J. (2001). Cortical axon guidance by the glial wedge during the development of the corpus callosum. *J. Neurosci.* 21 2749–2758. 10.1523/JNEUROSCI.21-08-02749.2001 11306627 PMC6762517

[B92] Silver J. Edwards M. A. Levitt P. (1993). Immunocytochemical demonstration of early appearing astroglial structures that form boundaries and pathways along axon tracts in the fetal brain. *J. Comp. Neurol.* 328 415–436. 10.1002/cne.903280308 8440789

[B93] Silver J. Lorenz S. E. Wahlsten D. Coughlin J. (1982). Axonal guidance during development of the great cerebral commissures: descriptive and experimental studies, in vivo, on the role of preformed glial pathways. *J. Comp. Neurol.* 210 10–29. 10.1002/CNE.902100103 7130467

[B94] Silver J. Ogawa M. Y. (1983). Postnatally induced formation of the corpus callosum in acallosal mice on glia-coated cellulose bridges. *Science* 220 1067–1069. 10.1126/science.6844928 6844928

[B95] Sival D. A. Guerra M. Den Dunnen W. F. A. Bátiz L. F. Alvial G. Castañeyra-Perdomo A. (2011). Neuroependymal denudation is in progress in full-term human foetal spina bifida aperta. *Brain Pathol.* 21 163–179. 10.1111/J.1750-3639.2010.00432.X 21269337 PMC8094240

[B96] Smith K. M. Ohkubo Y. Maragnoli M. E. Rasin M.-R. Schwartz M. L. Sestan N. (2006). Midline radial glia translocation and corpus callosum formation require FGF signaling. *Nat. Neurosci.* 9 787–797. 10.1038/nn1705 16715082

[B97] Suárez R. Gobius H. Richards L. J. (2014). Evolution and development of interhemispheric connections in the vertebrate forebrain. *Front. Hum. Neurosci.* 8:497. 10.3389/FNHUM.2014.00497 25071525 PMC4094842

[B98] Taverna E. Götz M. Huttner W. B. (2014). The cell biology of neurogenesis: toward an understanding of the development and evolution of the neocortex. *Annu. Rev. Cell. Dev. Biol.* 30 465–502. 10.1146/ANNUREV-CELLBIO-101011-155801 25000993

[B99] Thomas S. Boutaud L. Reilly M. L. Benmerah A. (2019). Cilia in hereditary cerebral anomalies. *Biol. Cell* 111 217–231. 10.1111/BOC.201900012 31177551

[B100] Tramontin A. D. García-Verdugo J. M. Lim D. A. Alvarez-Buylla A. (2003). Postnatal development of radial glia and the ventricular zone (VZ): a continuum of the neural stem cell compartment. *Cereb. Cortex* 13 580–587. 10.1093/CERCOR/13.6.580 12764031

[B101] Unni D. K. Piper M. Moldrich R. X. Gobius I. Liu S. Fothergill T. (2012). Multiple Slits regulate the development of midline glial populations and the corpus callosum. *Dev. Biol.* 365 36–49. 10.1016/j.ydbio.2012.02.004 22349628

[B102] Verhagen J. M. A. Schrander-Stumpel C. T. R. M. Krapels I. P. C. de Die-Smulders C. E. M. van Lint F. H. M. Willekes C. (2011). Congenital hydrocephalus in clinical practice: a genetic diagnostic approach. *Eur. J. Med. Genet.* 54:e542-7. 10.1016/j.ejmg.2011.06.005 21839187

[B103] Wagner C. Batiz L. F. Rodríguez S. Jiménez A. J. Páez P. Tomé M. (2003). Cellular mechanisms involved in the stenosis and obliteration of the cerebral aqueduct of hyh mutant mice developing congenital hydrocephalus. *J. Neuropathol. Exp. Neurol.* 62 1019–1040. 14575238 10.1093/jnen/62.10.1019

[B104] Wilkinson D. G. (1992). *In Situ Hybridization: a Practical Approach.* Oxford: IRL Press at Oxford University Press.

[B105] Wolman M. A. Sittaramane V. K. Essner J. J. Yost H. J. Chandrasekhar A. Halloran M. C. (2008). Transient axonal glycoprotein-1 (TAG-1) and laminin-alpha1 regulate dynamic growth cone behaviors and initial axon direction in vivo. *Neural Dev.* 3:6. 10.1186/1749-8104-3-6 18289389 PMC2278142

[B106] Yoo S. W. Motari M. G. Schnaar R. L. (2017). Agenesis of the corpus callosum in Nogo receptor deficient mice. *J. Comp. Neurol.* 525 291–301. 10.1002/CNE.24064 27339102 PMC5138123

[B107] Yoon T. Y. Munson M. (2018). SNARE complex assembly and disassembly. *Curr. Biol.* 28 R397–R401. 10.1016/J.CUB.2018.01.005 29689222

