# Supplementary material for: A selective defect in the glial wedge as part of the neuroepithelium disruption in hydrocephalus development in the mouse hyh model is associated with complete corpus callosum dysgenesis
Source: Front Cell Neurosci. 2024 Feb 21;18:1330412. doi: 10.3389/fncel.2024.1330412 (PMC10915275; doi:10.3389/fncel.2024.1330412)
Supplement: Supplementary file 1 [file Data_Sheet_1.PDF]

Supplementary Figure 1

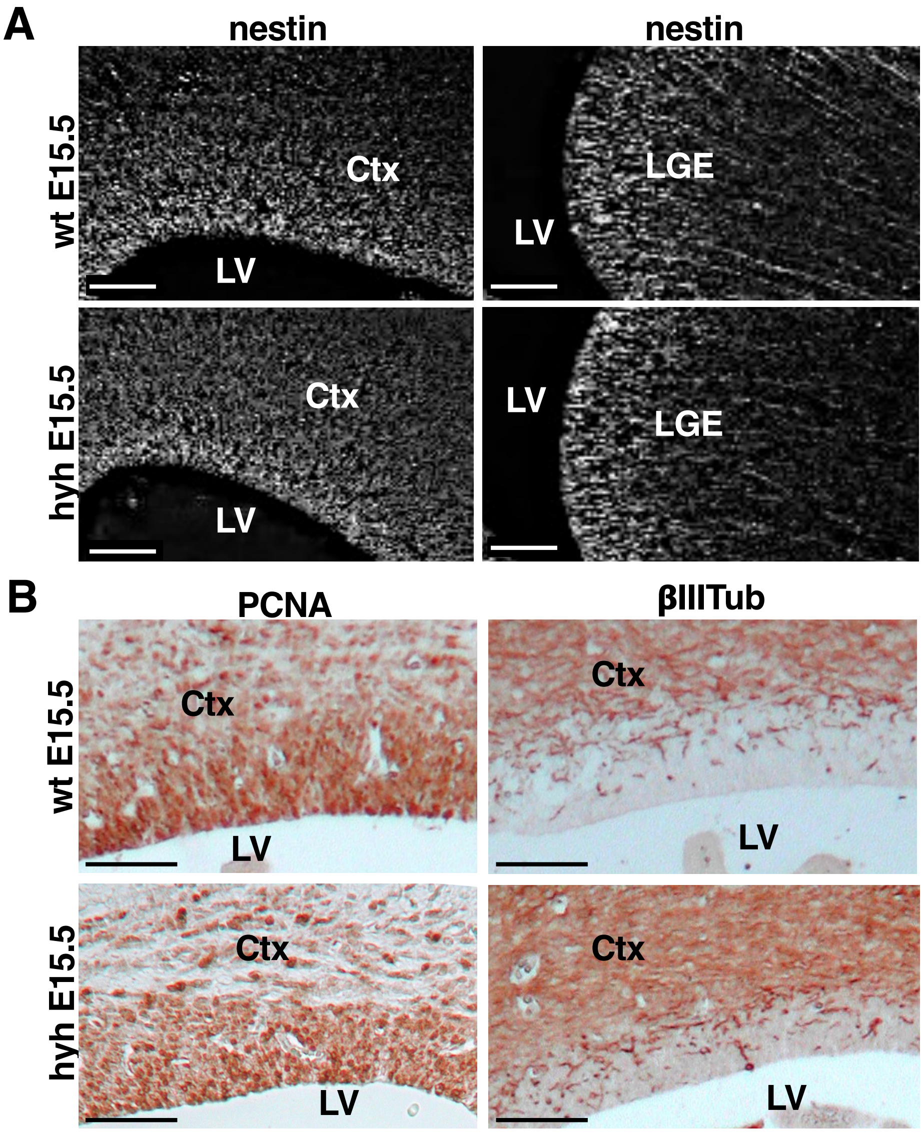

Ventricular zone areas are not altered in the hyh mice.
A, Frontal sections of wild-type (wt) (top) and hyh (bottom) mice brain at E15.5 immunostained with anti- Nestin, showing Dorsal Pallium on the left and Lateral Ganglionic Eminence on the right. **B**, Frontal sections of the Dorsal Pallium of wild-type (wt) (top) and hyh (bottom) mice brain at E15.5 immunostained with anti- PCNA (left) and βIII-Tubulin (right). Abbreviations: Ctx, cortex; LGE, Lateral Ganglionic Eminence; LV, Lateral Ventricle. Scale Bars:100μm
